# Water Co‐Adsorption in Ultrathin Films of Ionic Liquids on Pt(111)

**DOI:** 10.1002/open.202500571

**Published:** 2025-11-26

**Authors:** Timo Talwar, Hans‐Peter Steinrück, Florian Maier

**Affiliations:** ^1^ Lehrstuhl für Physikalische Chemie 2 Friedrich‐Alexander‐Universität Erlangen‐Nürnberg Erlangen Germany

**Keywords:** ARXPS (angle resolved X‐ray photoelectron spectroscopy), coadsorption, ionic liquids, SCILL (solid catalyst with ionic liquid layer), water

## Abstract

The influence of pre‐adsorbed water (D_2_O) on the growth, adsorption, orientation, and thermal behavior of two ionic liquids (ILs) on Pt(111) was studied within the SCILL (solid catalyst with ionic liquid layer) concept. Nonfunctionalized [C_1_C_1_Im][Tf_2_N] and nitrile‐functionalized [C_3_CNC_1_Im][Tf_2_N] were deposited at ∼100 K onto clean Pt(111) or onto single‐layer and multilayer crystalline (CI) or amorphous (ASW) D_2_O films and analyzed at various temperatures by angle‐resolved X‐ray photoelectron spectroscopy (ARXPS). On both clean and D_2_O‐covered Pt(111), the ILs initially grow in a 2D layer‐by‐layer mode, forming a closed wetting layer at ≈ 0.5 ML IL coverage, followed by moderate 3D growth above 0.8–1.0 ML. At 100 K, the ILs partially displace D_2_O from the Pt surface, yielding co‐adsorption structures where IL contacts Pt(111) directly and is also located in a second layer. [Tf_2_N]^−^ anions adsorb in cis‐configuration with oxygen atoms toward the surface, while the cations adopt mixed parallel and tilted orientations. Heating to 130 K induces rearrangement, increasing direct IL–Pt(111) contact by favoring parallel cation alignment. Co‐adsorbed D_2_O remains but desorbs at ≈10 K lower temperature, indicating weaker binding to Pt. The similar interfacial behavior of both ILs shows that the nitrile group does not significantly influence adsorption geometry or thermal stability.

## Graphical abstract

At 100 K, IL adsorption on D_2_O precovered Pt(111) partially displaces interfacial water resulting in a water‐IL co‐adsorbed layer. Upon heating to 130 K, IL ions migrate to the metal interface, thereby destabilizing adsorbed water as shown by the ≈10 K reduction in its desorption temperature.

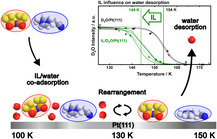

## 1 Introduction

Ionic liquids (ILs) are low‐melting salts typically composed of organic cations—such as imidazolium, pyridinium, or ammonium derivatives—and a wide variety of organic or inorganic anions. Their bulky structure and delocalized charge distribution decrease the strength of electrostatic interactions, preventing the formation of a stable crystal lattice and resulting in melting points below 100°C, often even below room temperature. Due to their ionic nature, ILs possess a suite of unique physicochemical properties, including negligible vapor pressure [1], wide electrochemical and thermal stability windows [2, 3]. Moreover, their chemical structure can be finely tuned by modifying either the cation or anion, enabling the design of task‐specific ILs for targeted applications in materials science, electrochemistry, separations, and catalysis [4, 5, 6].

In the latter, the solid catalyst with ionic liquid layer (SCILL) concept has gained attention for its ability to tune the activity and selectivity of a solid metal catalyst by applying an ultrathin layer of ionic liquid [7, 8, 9]. These films can modify the surface chemistry through a variety of mechanisms, including site blocking, selective adsorption of reactants or products, stabilization of charged or polar intermediates, and modification of the local electric field at the interface. SCILL‐modified systems have shown improved selectivity in hydrogenation reactions [6, 8, 10, 11, 12, 13], electrochemical CO_2_ reduction [14], and oxidation reactions [15].

The detailed structure and dynamics at the IL–solid interface play a central role in determining the overall catalytic performance. To rationally design SCILL systems, a molecular‐level understanding of IL–metal interactions is essential. This is typically achieved by studying well‐defined model systems, in which single‐crystal metal surfaces are modified with ultrathin IL films under ultrahigh vacuum (UHV) conditions. These films are usually prepared via physical vapor deposition (PVD), and their growth, adsorption/orientation, and thermal stability are characterized using surface science techniques such as AFM/STM, IRAS, and XPS [16, 17, 18, 19, 20, 21, 22, 23, 24, 25, 26, 27, 28, 29, 30, 31]. Model studies frequently involve imidazolium‐based ILs, such as [C_8_C_1_Im]^+^ or [C_1_C_1_Im]^+^, paired with anions like [Tf_2_N]^−^, [TfO]^−^, [BF_4_]^−^ or [PF_6_]^−^ on surfaces including Au(111) [16, 22, 23, 24, 32, 33, 34], Ni(111) [35], Ag(111) [25, 26, 29, 30], Cu(111) [20, 33], Pt(111) [17, 18, 21, 36, 37, 38], and various metal oxides [35, 39, 40].

A major challenge in advancing SCILL systems lies in understanding how common molecular co‐adsorbates—such as water—influence interfacial structure and function. Water is frequently present in catalytic systems as a reactant, product, or impurity and can interact with both the IL and the metal surface, thereby affecting adsorption equilibria, interfacial dynamics, and catalytic pathways. The presence of water may lead to restructuring of the IL layer, solvation or displacement of adsorbed species, or even promote electron transfer processes. Characterizing how water co‐adsorbs and distributes within IL films on Pt(111) is therefore essential for understanding and optimizing SCILL performance under realistic conditions.

In this study, we investigate the co‐adsorption of deuterated water (D_2_O) with two ILs—1,3‐dimethylimidazolium bis(trifluoromethyl‐sulfonyl)imide ([C_1_C_1_Im][Tf_2_N]) and 1‐(3‐cyanopropyl)‐3‐ methylimidazolium bis(trifluoromethyl‐sulfonyl)imide ([C_3_CNC_1_Im][Tf_2_N])—on a Pt(111) surface under ultrahigh vacuum (UHV) conditions. We employed D_2_O instead of H_2_O, in order to prepare for thermal desorption studies with D_2_O planned in the future. The latter IL incorporates a polar nitrile group, distinguishing it from the nonfunctionalized [C_1_C_1_Im][Tf_2_N]. Previous studies have shown that this functionalization enhances thermal stability on Pt(111) in the absence of water [36]. Here, we explore how this structural difference affects co‐adsorption with water. Angle‐resolved X‐ray photoelectron spectroscopy (ARXPS) is employed to probe the growth mode and thermal behavior of both ILs on a D_2_O pre‐covered Pt(111) surface. The results are compared to those from neat IL and neat D_2_O adsorption, allowing us to assess the influence of the IL and the IL structure—and specifically the functional group—on interfacial water behavior.

## 2 Experimental

ARXPS measurements were performed using a two‐chamber XPS setup with a non‐monochromatized Al Kα X‐ray source (SPECS XR 50, 1486.6 eV, 240 W) and a hemispherical electron analyzer (VG SCIENTA R3000). The base pressure in the analysis chamber was ≈3 × 10^−11^ mbar and in the preparation chamber ≈3 × 10^−10^ mbar. Spectra were recorded with a pass energy of 200 eV, resulting in a resolution of ≈ 1 eV [21]. The Pt(111) circular crystal was purchased from MaTeck (15 mm in diameter, purity 99.999 %, polished with an alignment better than 0.1° to the (111) plane), the IL [C_1_C_1_Im][Tf_2_N] was bought from Iolitec (purity 99.5%), and the D_2_O (99.96 at% D) from Aldrich. The synthesis of the IL [C_3_CNC_1_Im][Tf_2_N] was described elsewhere [41]. The Pt(111) surface was cleaned by Ar‐sputtering (600 V) for 30 min, followed by an annealing step at 1100 K for 10 min, then at 800 K for 10 min in an oxygen atmosphere of 10^−7^ mbar, and finally the remaining oxygen was desorbed by flashing to 1000 K for 10 s in ultra‐high vacuum. The cleanliness of the surfaces was checked by XPS. The two custom‐built Knudsen‐cell‐type evaporators were filled with [C_1_C_1_Im][Tf_2_N] and [C_3_CNC_1_Im][Tf_2_N] and subsequently degassed for multiple hours at elevated temperature to eliminate any residual volatile impurities. The purity of the ILs was checked by XPS of macroscopic IL films; all core level intensities agreed with the nominal composition to within 5%. D_2_O was placed in a glass‐tube, degassed by multiple cycles of freeze pumping and dosed from background through a leak‐valve system at 2–5 × 10^−9^ mbar in the preparation chamber. The mass spectrum during dosing showed the expected signals, and the XPS spectrum showed only the O 1*s* signal of D_2_O; in particular, there was no indication of adsorbed carbon species.

On Pt(111), an ordered first layer of D_2_O can be prepared by dosing at temperatures above ≈130 K, which is the seed layer for the growth of crystalline ice (CI) in the multilayer regime. In contrast, dosing below ≈110 K results in a disordered first layer that is the seed layer for amorphous solid water (ASW) in the multilayer regime (see Figure S1a in the Supporting Information). In this work, the terms CI and ASW are also used to describe the structure of the first water layer. To prepare a CI film, a D_2_O multilayer was dosed onto Pt(111) at 137–140 K. At this temperature, the D_2_O multilayer slowly desorbs, resulting in a saturated wetting layer, which showed a faint (√3 × √3)R30° LEED pattern (see Figure S1), which disappeared upon electron irradiation. The presence of the D_2_O layer was confirmed by XPS and the sample was cooled to 100 K. ASW films were prepared by dosing D_2_O on Pt(111) at 100 K; for this layer, no ordered adsorbate‐induced LEED pattern was observed. The coverage is given in wetting layers (WL), where 1 WL corresponds to the O 1*s* intensity of a fully saturated CI layer on Pt(111). After prolonged X‐ray exposure, minor beam‐induced D_2_O desorption could be observed.

For IL deposition, the IL evaporators were preheated to 453 K for [C_3_CNC_1_Im][Tf_2_N], or 398 K for [C_1_C_1_Im][Tf_2_N]; the ILs were then dosed to the clean or the D_2_O precovered Pt(111) surface at 100–110 K. Film thicknesses were determined from the exponential attenuation of the substrate signal by the covering IL film, using the inelastic mean free path of 3.1 nm for Pt 4*f* electrons, as described in previous publications [21]. The Pt 4*f* area was obtained by numerical integration of the Pt 4*f*_5/2_ and 4*f*_7/2_ region from 66.65 to 76.85 eV after subtraction of a linear background, as described in the Supplementary Information to Ref. [18]. The IL film coverage is typically given in the units of monolayer (ML), which we define as a closed layer of anion and cation stacked vertically. In this definition, a fully covered surface, in which anions and cations are adsorbed in an alternating checkerboard‐like configuration—commonly referred to as the wetting layer (WL)—corresponds to a coverage of 0.5 ML. In this analysis, the density of the wetting layer at the metal interface is assumed to be equivalent to the bulk density of the ionic liquid ([C_3_CNC_1_Im][Tf_2_N]: 1.51 g/cm^3^; [C_1_C_1_Im][Tf_2_N]: 1.57 g/cm^3^) [42, 43], while neglecting surface‐related effects such as distinct adsorption geometries. For isothermal experiments, the IL was deposited at low temperature onto the D_2_O‐precovered Pt(111) surface and then heated to and held at the targeted temperature, while all IL‐relevant regions were recorded. For each temperature, a freshly prepared film was analyzed first at 0° and then at 80°. In temperature‐programmed XPS (TPXPS), the IL‐covered substrate was heated linearly with 2 K/min up to 600 K while sequentially measuring the Pt 4*f* signal of the substrate, the IL F 1*s* signal, and the overlapping O 1*s* signal from both IL and D_2_O. For reference measurements with pure D_2_O on Pt(111), only the Pt 4*f* and O 1*s* regions were monitored. To ensure a sufficient signal‐to‐noise ratio while minimizing beam‐induced damage, the scan rate and binding energy window were adjusted individually for each spectral region. In the growth experiments, the IL was deposited in multiple steps onto either clean or D_2_O‐pre‐covered Pt(111) at 100 K. After each deposition step, the Pt 4*f* region was measured at emission angles of 0° and 80°.

The data was evaluated with CasaXPS V.2.3.25PR1.0. The spectra were referenced to the Pt 4*f*_7/2_ signal yielding 71.2 eV. At 0°, the F 1*s*, S 2*p* and N 1*s* regions were fitted with a linear background, the O 1*s* region with a cubic spline polynomial, and the C 1*s* region with a cubic spline polynomial (C_an_ peak) in combination with a linear background (C_cat_ peak). At 80°, the F 1*s*, C 1*s*, S 2*p* and N 1*s* region were fitted with a linear background, while for the O 1*s* region a Shirley background was used. All IL signals were fitted with a symmetric pseudo‐Voigt line shape (Gaussian 70% and Lorentzian 30%). The O 1*s* region consisted of overlapping contributions from D_2_O (O_D2O_) and IL (O_an_). To deconvolute this region, the intensity of the O_an_ signal was first determined by referencing the F 1*s* (F_an_) signal of the IL, using a fixed intensity ratio of F 1*s* to O 1*s* (F_an_/O_an_) of 2.8, as derived from averaging the ratios of the neat ILs on Pt(111) (see Supporting Information). The adsorbed water was represented by the O_D2O_ peak and to account for possible water desorption, the intensity could vary freely. The full width at half maximum (FWHM) for each peak was held constant for coverages up to 0.5 ML IL and 1 WL D_2_O, irrespective of temperature. The applied values are summarized in Table S1.

Due to the lower signal to noise ratio, the O 1*s* signals in the TPXP spectra were not deconvoluted into individual O_an_ and O_D2O_ components. Instead, the O 1*s* region was fitted with a single symmetric peak, using a variable FWHM ranging from ∼1.8 eV (anion signal O_an_ of neat ILs) to 2.1 eV (combined O_an_ + O_D2O_) to better accommodate signal changes as a function of temperature. For the F_an_ component, the same FWHM were applied as listed in Table S1. In the multilayer TPXPS spectra, that is, for IL coverages >0.5 ML and D_2_O coverages >1 WL, the FWHM is expected to deviate from the wetting layer coverages, due to the superposition of interface and bulk contributions to the signals, and consequently, no constraints were applied.

In some cases, the Pt(111) showed a residual carbon impurity in the C_cat_‐region after the standard cleaning procedure. This impurity signal was scaled down according to the attenuation of the substrate signal and accounted for when fitting the IL‐spectra (for more information on this correction, see Supplementary Information to Ref. [20]). When comparing to data from previous studies [21, 36], the absolute intensities were scaled using the spectra of the clean Pt(111) sample, to account for changes in photon flux and detector sensitivity.

## 3 Results and Discussion

### 3.1 Growth of [C_1_C_1_Im][Tf_2_N] and [C_3_CNC_1_Im][Tf_2_N] on D_2_O/Pt(111)

The growth behavior of [C_1_C_1_Im][Tf_2_N] and [C_3_CNC_1_Im][Tf_2_N] on D_2_O/Pt(111) was monitored through the attenuation of the Pt 4*f* core level as a function of the IL coverage. For perfect homogeneous 2D growth, the Pt 4*f* signal, $I_{d}/I_{d=0}$, should decrease exponentially with increasing IL film thickness *d* according to (Equation 1):

(1) $$I_{d}/I_{d=0}=\exp\;(-d/\lambda \cos\vartheta )$$

with λ being the inelastic mean free path of the Pt 4*f* electrons within the IL (3.1 nm) [21, 29] and ϑ the emission angle of the electrons with respect to the surface normal. Notably, in ideal layer‐by‐layer growth, the Pt 4*f* signal decreases linearly within each layer, and the completion of each full layer results in a stepwise exponential decrease. While the resolution of our data does not allow us to clearly identify individual slope changes between consecutive layers, the overall growth mode can still be determined by comparing signal attenuation at different emission angles. Following the procedure from previous studies [27], the mean film thickness *d* was first calculated from the experimental $I_{d}/I_{d=0}$ ratio obtained at ϑ = 0°, that is, the bulk‐sensitive configuration. Using this value and (Equation 1), the expected attenuation at ϑ = 80° (surface‐sensitive) was calculated. A match between the experimental and calculated 80° values indicates uniform 2D growth. In contrast, experimental $I_{d}/I_{d=0}$ ratios exceeding the calculated 80° curve suggest the presence of a 3D morphology.

To apply this analysis on our system, we prepared the Pt(111) surface by dosing 1 WL D_2_O at 137–140 K (CI layer) on the freshly cleaned crystal (see Experimental), cooled it to 100 K, and subsequently deposited IL in multiple steps. After each step, the Pt 4*f* signal was measured at 0° and 80°. The same type of experiments was also done with the functionalized IL [C_3_CN_1_Im][Tf_2_N].

**FIGURE 1:**
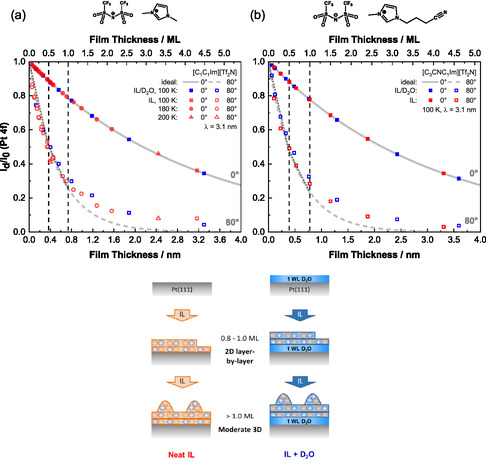
Growth of (a) [C_1_C_1_Im][Tf_2_N] and (b) [C_3_CN_1_Im][Tf_2_N] on clean Pt(111) (red symbols) and on 1 WL of crystalline (CI) D_2_O on Pt(111) (blue) as deduced from the attenuation of the Pt 4*f* intensity *I* *_d_*/*I*_0_ as a function of mean IL film thickness d in normal (0°, solid symbols) and in surface‐sensitive grazing (80°, open symbols) emission. The denoted temperatures refer to the respective IL deposition and measurement temperature. Note that *I*_0_ is the Pt intensity prior to the first IL deposition step, that is, either measured for the clean crystal (red) or for the crystal already covered by the D_2_O WL (blue). The solid and dashed gray lines show the exponential decay expected for 2D layer‐by‐layer growth for emission angles of 0° and 80°, respectively; they are calculated using Equation (1) with inelastic mean free paths *λ* of 3.1 nm for Pt 4*f* electrons. The axis at the top of the figures denote the thickness in IL monolayers (ML); the black dashed vertical lines indicate 0.5 and 1.0 ML. At the bottom of the figure a schematic sketch of the growth behavior is depicted. For details, see text. The data from [C_1_C_1_Im][Tf_2_N] on clean Pt(111) ((a) red datapoints) is adapted from Ref. [21] with permission from PCCP Owner Societies.

We start by discussing the growth of the non‐functionalized IL [C_1_C_1_Im][Tf_2_N]. The results are shown in Figure 1a (note that the mean film thickness *d* is provided as bottom and top *x*‐axis in nm and in IL ML, respectively). The red data points represent the IL (adapted from Ref. [21]) deposited on Pt(111) without pre‐dosed D_2_O, and the blue data points the IL on top of D_2_O/Pt(111). Different temperatures are represented by distinct symbols. Closed and open symbols represent data measured at 0° and 80°, respectively. The exponential attenuations at 0° and 80° (expected for uniform 2D growth) are shown by grey solid and dashed lines, respectively. The two vertical dashed lines mark coverages of 0.5 and 1.0 ML, that is, a fully saturated IL wetting layer and the first multilayer on top, respectively. The data points for [C_1_C_1_Im][Tf_2_N] on D_2_O/Pt(111) (blue open symbols) match the ideal 2D curve (grey dashed line) up until ≈1.0 ML, and at higher coverages, they systematically deviate from the ideal curve up until ≈4.5 ML. We interpret this as indicated by the scheme at the bottom of Figure 1a: The first layer, that is, up to 0.5 ML, grows in a 2D layer‐by‐layer fashion. In the second layer, initially the 2D growth continues and around ≈1 ML (2 WL) it switches to moderate 3D. The growth behavior resembles that of the neat [C_1_C_1_Im][Tf_2_N] on Pt(111) (red data points), which also proceeds in a 2D mode up to 0.8–1.0 ML before transitioning to moderate 3D growth at higher coverages. Altogether, the presence of the water layer appears to have only a minimal influence on the growth of [C_1_C_1_Im][Tf_2_N] on Pt(111).

Next, we examine the data of the nitrile‐functionalized IL [C_3_CNC_1_Im][Tf_2_N], shown in Figure 1b. For the neat IL (red open symbols), the data again follows the ideal 2D curve up to 0.8–1.0 ML, and the same behavior is also seen for [C_3_CNC_1_Im][Tf_2_N]/D_2_O/Pt(111) (blue open symbols) within the experimental uncertainty. Both systems exhibit moderate 3D growth at higher coverages. Interestingly, the nitrile‐functionalized IL [C_3_CNC_1_Im][Tf_2_N] on clean Pt(111) at 100 K exhibits a very similar behavior as on Au(111) at 150 K, with 2D growth up until 0.75 ML, followed by moderate 3D growth [36].

### 3.2 Thermal Evolution of D_2_O on Pt(111)

**FIGURE 2:**
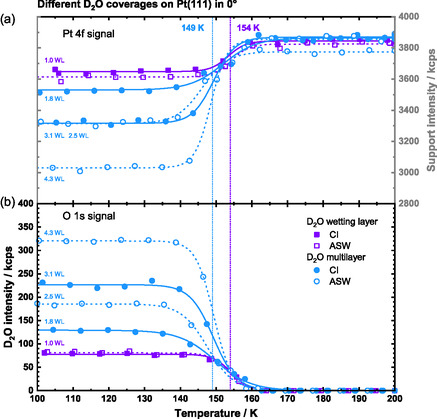
TPXPS of different water (D_2_O) coverages on Pt(111). (a) Pt 4*f* and (b) O 1*s* (D_2_O) signal. The water layers have been prepared either at 100 K (ASWlayer) or at 137−140 K (CI layer); for details see text. The violet symbols and lines indicate the coverage of ≈1 WL (wetting layer) while the blue symbols and lines indicate coverages in the multilayer range. The vertical dotted lines indicate the desorption temperature of 149 K for the wetting layer and 154 K for the multilayer, as determined from the inflection points (IP).

After investigating the growth behavior of both ILs on top of D_2_O/Pt(111), we focus on the thermal stability of the deposited films on Pt(111) in the following. For comparison, we start with neat D_2_O layers alone on Pt(111). Different D_2_O coverages of CI and ASW films were prepared, cooled to 88–105 K, and heated with a constant ramp of 2 K/min while continuously recording the Pt 4*f* and O 1*s* regions. In the O 1*s* region, a spline polynomial background was applied for background subtraction to remove the tail of the Pt 3*p*_3/2_ peak. The background was parameterized using the O 1*s* region measured for the clean Pt(111) crystal. These parameters fit well for low coverages such as 0.5 ML IL or 1.0 WL D_2_O, but introduce a small error at higher coverages, due to increased attenuation of the underlying Pt signal. This background type was nevertheless retained without further adjustment for higher coverages, as the associated error remained minimal (estimated ≈1% for the maximum 4.3 WL D_2_O coverage film) and did not significantly affect the analysis. Figure 2 displays the integrated signals of the temperature programmed XP‐spectra of D_2_O: purple symbols represent single CI and ASW D_2_O wetting layers, while blue symbols represent multilayer films. Open and closed symbols, along with dashed and solid lines, correspond to ASW and CI films, respectively. We start by analyzing the wetting layer films (1.0 ± 0.1 WL, purple lines/symbols). Both the ASW and CI film decrease to zero intensity at ≈154 K (IP = inflection point) in the O 1*s* region, while their corresponding Pt 4*f* signal increases at ≈153 K (IP) due to the decreased attenuation. These desorption temperatures align well with the reported wetting layer desorption temperature of 160–168 K [44, 45], taking our very slow heating ramp into account. Using a simple Redhead analysis for first order desorption with a prefactor of 3.1 × 10^15^ s^−1^ from Ref. [45], the desorption temperature of 154 K corresponds to a desorption energy of 51.8 kJ/mol [46]. In the D_2_O multilayer TPXPS (blue lines/symbols), a single desorption step, at 149 ± 3 K (IP; Pt 4*f* and O 1*s* signal), is observed, too, corresponding to a desorption energy of 50.1 kJ/mol. The presence of only one step instead of two is attributed to the small difference of wetting layer and multilayer desorption temperatures, which does not allow for resolving the two subsequent desorption steps in the (integrating) TPXPS experiment. According to temperature programmed desorption studies in literature, multilayer desorption of water from Pt(111) at comparable coverages typically occurs ≈10 K below the wetting layer desorption temperature [44]. The average of the inflection points of the multilayer films is ≈149 K, which is ≈5 K lower than the wetting layer desorption at ≈154 and ≈5 K higher than the expected multilayer desorption. Note that the IP for thicker multilayers is slightly shifted to higher temperatures, as is commonly observed for zero‐order desorption processes.

### 3.3 [C_1_C_1_Im][Tf_2_N] on D_2_O/Pt(111)

#### 3.3.1 Thermal Evolution

**FIGURE 3:**
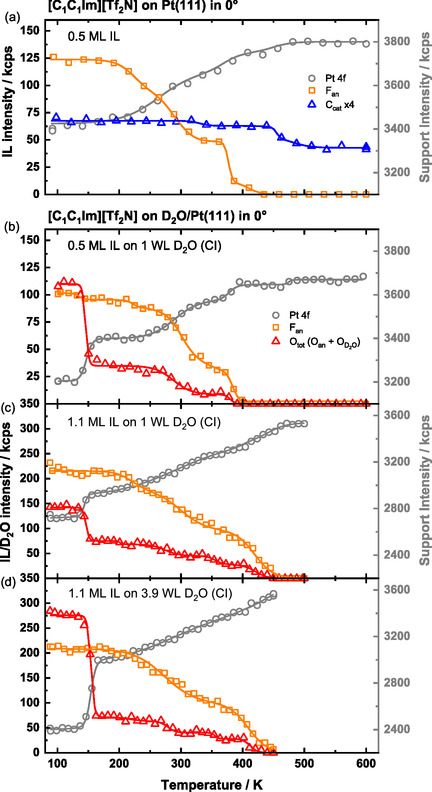
TPXPS of (a) neat 0.5 ML [C_1_C_1_Im][Tf_2_N] (adapted from Ref. [21] with permission from PCCP Owner Societies) and (b–d) different co‐adsorbed [C_1_C_1_Im][Tf_2_N] and D_2_O coverages on Pt(111). The Pt 4*f* (grey circles), C 1*s* (blue triangles), O 1*s* (red triangles) and F 1*s* (orange squares) core levels were measured while heating the sample with a heating rate of 2 K/s from ≈100 up to 600 K. The intensity scale for the Pt 4*f* signals is shown on the right side. The F_an_/O_tot_ intensity ratio of (b–d) is plotted in Figure S2.

Prior to analyzing the thermal evolution of the IL on a D_2_O wetting layer on Pt(111), we examine the behavior of 0.5 ML [C_1_C_1_Im][Tf_2_N] on clean Pt(111) as a reference system. The corresponding TPXPS data, adapted from Ref. [21], was also measured with a heating rate of 2 K/min and is shown in Figure 3a. In this data set, the Pt 4*f* region (grey circles), the F 1*s* region (F_an_, orange squares), and the C 1*s* region (C_cat_, blue triangles) were measured, representing the support, the anion and the cation, respectively. The C_cat_ signal was enhanced by the factor of 4 for better visibility. As stated earlier [21], the IL WL remains stable between 100 and 200 K, after which partial decomposition begins. This is evidenced by a decrease in the F 1*s* signal, indicating desorption of anion‐derived products, while cation‐derived fragments remain adsorbed. The intensity plateau around 350 K indicates that the decomposition is slowed down due to passivation of the Pt(111) surface by decomposition products formed at the surface. At higher temperatures, further decomposition occurs, with complete desorption of the anion‐derived species. Most cation‐derived products remain as stable carbon residue up to at least 800 K.

Next, we address the TPXPS of 0.5 ML [C_1_C_1_Im][Tf_2_N] on 1 WL D_2_O (CI) on Pt(111). This composite layer corresponds to about 8 D_2_O molecules per IL ion pair (see Supporting Information). The sample was prepared by dosing D_2_O at 137–140 K, cooling to ≈100 K and depositing 0.5 ML IL on top. During the TPXPS measurement, the Pt 4*f*, F 1*s*, and O 1*s* signals were recorded sequentially. The C 1*s*, S 2*p* and N 1*s* regions were not measured and the scan rates were decreased to increase the signal‐to‐noise ratio of the remaining signals, while keeping the beam damage at a reasonable level. In Figure 3b, the integrated areas of the spectra are shown. The Pt 4*f* (grey circles) and F 1*s* (F_an_, orange squares) signals represent the substrate and the IL, respectively, while the O 1*s* signal (O_tot_, red triangles) includes contributions from both D_2_O and the IL anion. The O 1*s* region was not deconvoluted in the TPXPS series (this will only be done for isothermal experiments with better statistics; see below).

We start discussing the O_tot_ signal. At the beginning of the experiment (100 K) the signal is composed of the contribution of D_2_O (≈74 kcps, measured after D_2_O deposition) plus that of the IL (total: ≈108 kcps). Upon heating, the intensity decreases in three steps to zero. The first drop occurs at ≈146 K (IP) with a decrease of ≈73 kcps—closely matching the initial O_D2O_ intensity of 74 kcps of the predeposited D_2_O layer. This step is therefore attributed to the desorption of the D_2_O layer. However, considering the attenuation of about 15% by the post‐deposited IL overlayer remaining on top, one would expect an O_D2O_ intensity decrease only of ≈63 kcps. This difference suggests that the D_2_O layer is less attenuated by the IL than anticipated, that is, D_2_O is replaced at the Pt surface by IL in course of the deposition step even at 100 K. The remaining two steps in the O_tot_ signal at ≈288 K (IP) and ≈380 K (IP) are attributed to the decomposition of the remaining IL, consistent with the behavior of the neat IL F_an_ signal shown in Figure 3a.

We now turn to the F_an_ signal (Figure 3b), which also decreases in three steps. The relatively small first drop at ≈135 K is attributed to a rearrangement of the IL on the Pt(111) surface in the course of the desorption of D_2_O. In the following, the IL remains stable up to ≈200 K. The remaining steps in the F_an_ signal are attributed to IL decomposition similar to the neat IL; notably, a constant F 1*s*/O 1*s* ratio of 2.8 is observed between ≈160 and ≈270 K (see Figure S2), indicating a parallel decrease of these anion signals. The desorption of D_2_O (IP_Pt__4*f*_ = ≈147 K) and of IL decomposition products is further supported by the concurrent increase in the Pt 4*f* signal (grey circles) at these temperatures. For the interpretation, we will focus on the IL/D_2_O (O 1*s*) inflection points. TPXPS shows that in a 0.5 ML IL/1 WL D_2_O/Pt film, water desorbs 8 K earlier (at ≈146 K (IP)) compared with a 1 WL D_2_O/Pt film (∼154 K (IP), see Figure 2). This temperature shift indicates that the IL destabilizes the water film on Pt(111) and promotes rearrangement into a second layer, since the desorption temperature approaches that of neat multilayer D_2_O (≈149 K (IP)).

To investigate whether the water desorption behavior changes with IL coverage, we post‐deposited 1.1 ML [C_1_C_1_Im][Tf_2_N] onto a D_2_O wetting layer (leaving the preparation procedure otherwise identical); see Figure 3c (note the different intensity scale compared to a and b). Starting with the O_tot_ signal, the first step occurs at ≈146 K (IP), with a decrease from 142 to 75 kcps, that is, by ≈67 kcps. This decrease is again smaller than the signal increase upon D_2_O deposition (75 kcps), but the signal is still higher than the expected O_D2O_ signal of ≈51 kcps for full attenuation by a 1.1 ML IL film. This behavior is again indicative of a nonlayerwise adsorption of the IL on the D_2_O film, but rather intermixing even at 100 K. As in the 0.5 ML IL /1 WL D_2_O system (Figure 3b), this step is attributed to D_2_O desorption and is accompanied by a rise in the Pt 4*f* signal at ≈144 K (IP). During this phase, the F_an_ signal remains unchanged, indicating that the IL layer is unaffected by the D_2_O desorption. Above ≈190 K, both the F_an_ and O_tot_ signals begin to decrease continuously, consistent with the thermal behavior of the neat IL multilayer (not shown) and attributed to decomposition with ongoing exchange and desorption processes [21]. We therefore conclude that an increase in IL coverage does not influence the desorption temperature of water.

In the next step, the predosed water coverage was increased from 1.0 to 3.9 WL, while the post‐dosed IL coverage remained at 1.1 ML. The corresponding results are shown in Figure 3d. The O_tot_ signal now decreases by ≈204 kcps at ≈154 K (IP), which is again attributed to water desorption. The initial O_D2O_ intensity (prior to IL deposition) was ≈285 kcps, and the expected attenuation by ≈197 kcps is little smaller than the observed drop, but within the experimental uncertainty. The Pt 4*f* intensity increases simultaneously at ≈154 K (IP). Compared with the 1.1 ML IL/1.0 WL D_2_O system, the desorption temperature is shifted by ≈8 K to higher values. This shift is consistent with expectations for zero‐order multilayer desorption, where increased D_2_O coverage leads to a higher desorption maximum. Remarkably, the observed temperature is still ≈5 K higher than that of neat 4.3 WL D_2_O on Pt(111), which desorbs at ≈149 K (IP), suggesting that additional effects—such as IL‐induced stabilization of the water layer—may also contribute to the temperature shift. Above ≈190 K, a continuous parallel decrease in the F_an_ and O_tot_ signal is again observed, as previously described for the 1.1 ML film/1.0 WL D_2_O system (Figure 3c), and interpreted accordingly.

#### 3.3.2 Quantitative Analysis of 0.5 ML [C_1_C_1_Im][Tf_2_N] on 1 WL D_2_O/Pt(111)

**FIGURE 4:**
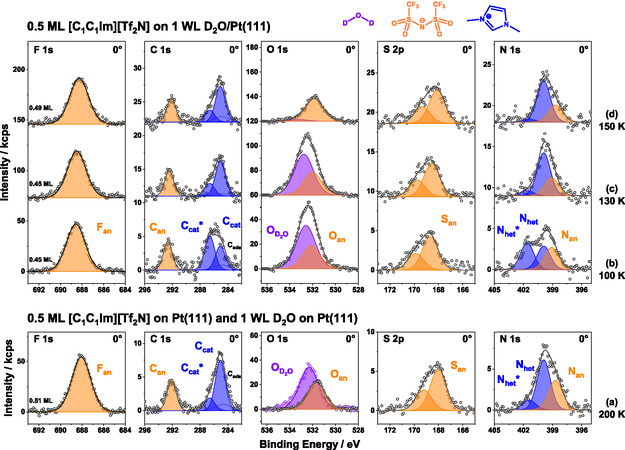
Isothermal XP‐spectra of 0.5 ML [C_1_C_1_Im][Tf_2_N] on (a) Pt(111) at 200 K (adapted from Ref. [21] with permission from PCCP Owner Societies) and on 1 WL D_2_O on Pt(111) at (b) 100, (c) 130, and (d) 150 K, measured at 0° emission. For each temperature a new sample was prepared. The peaks are color‐coded with blue for IL cation, orange for IL anion, and purple for D_2_O‐related signals. In (a), the O 1*s* spectrum of pure water is added for comparison (purple datapoints). It is worth noting that the spectra of the pure IL layer in (a) measured at 200 K are very similar to those measured at 100 K, apart from minor peak shifts (see Ref. [21]).

To gain deeper insight into the thermal behavior of [C_1_C_1_Im][Tf_2_N] on 1 WL of D_2_O/Pt(111), we measured all 5 IL‐related core levels at 100, 130, and 150 K and performed a detailed quantitative analysis. For each temperature, a new film was prepared using the following procedure: (1) cleaning Pt(111) crystal, (2) dosing D_2_O at 137–140 K to obtain a 1.0 ± 0.1 WL CI film (multilayer desorbs at this temperature), (3) depositing IL while cooling from 110 to 100 K, and (4) heating to the targeted temperature and recording a set of spectra at 0° and 80°. The 0° results are presented in Figure 4. The nominal IL coverage was 0.5 ML, with exact values of 0.45 ML for 100 and 130 K, and 0.49 ML for 150 K, as displayed in the F 1*s* region. The spectra are arranged in the measurement sequence. As a reference, the spectra of 0.5 ML neat [C_1_C_1_Im][Tf_2_N] on Pt(111) at 200 K were adapted from Ref. [21] (Figure 4a). Notably, a neat IL film annealed at 200 K (rather than 100 K) has been used as reference, since STM showed a higher degree of order and uniformity at this temperature of this film as compared with 100 K [21].

We begin discussing the data of [C_1_C_1_Im][Tf_2_N] on 1 WL of D_2_O/Pt(111) at 100 K (Figure 4b); the corresponding peak positions and full width at half maximum (FWHM) values are listed in Tables 1 and S1, respectively. The orange‐colored anion‐related components are the F 1*s* (F_an_) and C 1*s* (C_an_) signals of the CF_3_ group at ≈688.6 and ≈292.7 eV, respectively, the O 1*s* (O_an_) and S 2*p*_3/2_ (S_an_) signals of the SO_2_ group at ≈532.1 and 168.7 eV, respectively, and the N 1*s* signal of the imidic nitrogen (N_an_) at ≈399.1 eV. The dark blue colored cation‐related components are, the two C 1*s* signals, C_cat_* and C_cat_, at ≈286.6 and ≈285.1 eV, respectively, and the two N 1*s* signals, N_het_* and N_het_, at ≈401.6 and ≈399.9 eV, respectively, which all correspond to the imidazolium ring. The remaining purple‐colored O 1*s* (O_D2O_) signal at ≈532.6 eV corresponds to D_2_O. Since both oxygen signals are very close in binding energy, the fitted peak positions of O_D2O_ and O_an_ have some ambiguities due to limitations of the O 1*s* deconvolution process (see Experimental section), which was performed with the O_an_ intensity being constrained relative to the F_an_ signal using a fixed F_an_/O_an_ intensity ratio of 2.8 (deduced from XP spectra of the 0.5 ML IL layer on clean Pt(111)). Therefore, the O_an_ and O_D2O_ peak positions will not be discussed in detail.

**TABLE 1:** Peak positions for wetting and sub‐wetting layer coverages, that is, ≤0.5 ML IL and ≤1 WL D_2_O, of neat IL, IL/D_2_O (CI), and neat D_2_O (CI). Values marked with # are adapted from Ref. [21] with permission from the PCCP Owner Societies and with § are adapted with permission from Ref. [36]. Copyright 2024 American Chemical Society. For details see text.

Binding energy	F_an_	C_an_	C_cat_*	C_cat_	O_an_	S_an_	N_het_*	N_het_	N_an/CN_/N_an_	O_D2O_
D_2_O (CI)										
140 K	—	—	—	—	—	—	—	—	—	532.3
**[C_1_C_1_Im][Tf_2_N]/D_2_O**										
**Neat IL, 200 K^#^**	688.1	292.1	286.6	285.1	531.7	168.0	401.4	399.9	398.7	—
**Neat IL, 100 K^#^**	688.2	292.2	286.9	285.4	532.0	168.3	401.5	400.0	398.8	—
**150 K**	688.3	292.2	286.6	285.1	531.8	168.1	401.4	399.9	398.7	533.3
**130 K**	688.5	292.5	286.6	285.1	532.0	168.6	401.6	399.9	399.1	532.8
**100 K**	688.6	292.7	286.6	285.1	532.1	168.7	401.6	399.9	399.1	532.6
**[C_3_CNC_1_Im][Tf_2_N]/D_2_O**										
**Neat IL, 150 K** ^§^	688.3	292.3	286.4	285.3	531.9	168.3	401.4	400.2	399.0	—
**150 K**	688.3	292.3	286.5	285.3	531.8	168.3	401.4	400.2	399.0	532.4
**130 K**	688.6	292.5	286.5	285.3	532.2	168.7	401.7	400.2	399.2	532.7
**100 K**	688.7	292.7	286.5	285.3	532.0	168.7	401.7	400.2	399.5	532.6

All anion‐related peaks (except O_an_) of the 0.5 ML IL/1 WL D_2_O film are shifted toward higher binding energy relative to the peak positions of the neat IL WL on Pt(111); these shifts amount to 0.41–0.65 eV at 100 K; for O_an_, no reliable shift could be determined because of the denoted ambiguities with peak fitting due to the overlapping O_D20_ signal. The shifts might be related to IL‐water interactions (e.g., hydrogen bonds or dipole interactions); more likely, the localization of the IL anions further away from the Pt surface leads to a decreased core‐hole screening—and thus, higher binding energies—due to the presence of D_2_O. The smaller shift at 130 K points to the onset of the replacement of D_2_O at the interface by IL ions in the course of the above discussed water desorption step at 146 K. Before we are able to discuss the cation‐related signals of the IL/D_2_O film, we have to address the peak fitting of the neat IL on Pt(111) (further details, see also Ref. [21]): Both cationic signals, N_het_ and C_cat_, are fitted with two peaks each. One corresponding to IL in direct contact with the Pt(111) surface (N_het_ and C_cat_) and the other to IL in less favorable (multilayer‐like) adsorption sites (marked with an asterisk: N_het_* and C_cat_*). The first are shifted to lower binding energy due to more efficient core hole screening and the strong interaction of the *π*‐orbitals of the aromatic system with the Pt(111) *d*‐band [21]. In contrast to previous publications, two distinct peaks (with independent intensities) were used instead of a single asymmetric peak (composed of two peaks with constraint intensities) to more clearly resolve temperature‐dependent changes. For the asymmetric C_cat_ signal in Ref. [21], the two peaks correspond to the shoulder (C_cat_*) and primary peak (C_cat_). In the D_2_O/IL spectra, the binding energies of C_cat_*/C_cat_ components were constrained to the neat IL positions at 200 K [21]. The same approach is used for N_het_ (note: N_het_ is labeled N_cat_ in Ref. [21]). For the D_2_O/IL spectra at 100 and 130 K, the shoulder peak position (N_het_*) is shifted to even higher BE. In the spectra of the neat IL at 200 K (Figure 4a), the C_cat_*/C_cat_ and N_het_*/N_het_ ratios are 0.25 and 0.20, respectively. These ratios are strongly increased to ≈1.42 (C_cat_*/C_cat_) and ≈1.13 (N_het_*/N_het_) in the IL/D_2_O spectra at 100 K (Figure 4b), indicating that a major fraction of the cations is now either positioned further from the surface (on top of D_2_O) or located in a less favorable adsorption site environment. The presence of a considerable fraction of cations still in direct contact with the Pt(111) surface indicates that the IL is not entirely adsorbed atop the D_2_O layer. Instead, the data support a mixed configuration: Some IL resides in direct contact with the metal substrate, while some remains on D_2_O or on top of the IL. This mixed configuration also explains why the D_2_O signal is less attenuated by the IL layer than expected, as discussed in the TPXPS measurements (see above). Alternatively, one can also imagine that the kinetic energy of the impinging IL is sufficient to reorganize the pre‐adsorbed D_2_O film at 100 K such that one obtains an ensemble of coexisting D_2_O clusters and IL clusters on the Pt(111) surface (with an average height corresponding to the nominal film thickness; see Figure S3). While we have no direct evidence for this behavior, it would also be consistent with our data and we thus cannot rule it out.

In the IL/D_2_O film at 130 K (Figure 4c), all anion‐related peaks (except O_an_) shift by up to −0.20 eV toward lower BE compared to the IL/D_2_O film at 100 K. Relative to the neat IL at 200 K, these peaks remain shifted to higher BE by 0.35–0.53 eV. In the cation‐related signals, a clear change in peak shape is observed: the C_cat_*/C_cat_ and N_het_*/N_het_ ratios are now ≈0.36 and ≈0.12, respectively, closely resembling the peak shape of the neat IL (Figure 4a). We interpret this as follows: Increasing temperature enhances the mobility of the adsorbates (IL and water), enabling a surface rearrangement. The IL now appears to be more or less completely adsorbed in direct contact with the Pt(111) surface, similar to its behavior without pre‐adsorbed water. However, the water molecules likely continue to interact with the IL, as indicated by the persistent shifts of the anion‐related peaks to higher binding energies.

At 150 K (Figure 4d), and thus above the desorption temperature of D_2_O found in TPXPS, all anion‐related peaks are shifted by up to −0.42 eV toward lower binding energy compared with the IL/D_2_O film at 130 K, with peak positions now closely matching those of the neat IL at 200 K. Nevertheless, a slight residual shift of up to 0.20 eV remains. The cation‐related signals remain largely unchanged, with the C_cat_*/C_cat_ and N_het_*/N_het_ ratios staying approximately constant at ≈0.34 and ≈0.07, respectively. Most notably, the water signal in the O 1*s* region has nearly vanished, with only a trace remaining—potentially attributable to noise. We conclude that at 150 K water has desorbed, and the remaining IL exhibits similar signal characteristics as the neat IL at 200 K.

After the qualitative analysis, we now turn to the quantitative evaluation of the data. Table 2 shows the relative composition of the three IL/D_2_O films at 100, 130 and 150 K, normalized to the total number of IL atoms, which is 22. For this analysis, the intensities of the C_cat_*/C_cat_ and N_het_*/N_het_ peak pairs were summed up. At 100 K, the relative composition agrees well with the nominal values within our experimental uncertainty of ±15%. It should be noted that for the quantitative analysis, peak areas were corrected using atomic sensitivity factors (ASF), which were determined from bulk films. Notably, in the analysis of thin films (thinner than the information depth of XPS), a systematic small error is introduced, where signals with lower kinetic energies and thus smaller inelastic mean free paths (IMFP) (e.g., F 1*s*) are enhanced, while signals with higher kinetic energy/ longer IMFP signals (e.g., S 2*p*) are decreased, see Ref. [36] for a detailed discussion. A correction term proposed therein was deliberately not applied to ensure better comparability with the neat IL data presented in Ref. [21]. When comparing the D_2_O/IL data to the neat IL at 100 K, the quantitative results align well, showing only small deviations in the O_an_ and C_cat_ signals.

**TABLE 2:** Normalized (to 22 atoms) relative IL composition of 0.5 ML [C_1_C_1_Im][Tf_2_N] films with 1 WL D_2_O on Pt(111) at 100, 130, and 150 K. For C_cat_* and C_cat_, as well as N_het_* and N_het_ only the sum of the two contribution is given.

Composition		F_an_	C_an_	C_cat_* + C_cat_	O_an_	S_an_	N_het_* + N_het_	N_an_	Σ	Σ_cation_	Σ_anion_
Nominal		6	2	5	4	2	2	1	22	7	15
**Pt(111), 0.5 ML, 1 WL D_2_O (CI)**											
**150 K**	**0°**	7.1	1.9	4.3	4.2	1.8	1.8	0.8	22	6.2	15.8
	**80°**	8.6	2.3	3.3	3.7	1.9	1.4	0.9	22	4.7	17.3
**130 K**	**0°**	7.1	2.0	4.2	4.2	1.8	2.0	0.8	22	6.2	15.8
	**80°**	8.9	2.5	3.1	3.0	2.0	1.5	1.1	22	4.5	17.5
**100 K**	**0°**	6.7	2.1	4.8	4.0	1.7	1.9	0.9	22	6.7	15.3
	**80°**	8.6	2.5	4.0	2.1	2.0	1.9	0.8	22	6.0	16.0

Between 130 and 150 K, only small changes in the IL composition are observed, and the relative composition remains consistent with both the 100 K data and the nominal values within the experimental uncertainty: nevertheless, the small increase of the F_an_ signal (from 6.7 to 7.1) and the small decrease of the C_cat_* + C_cat_ signal (from 4.8 to 4.3) between 100 and 150 K are a further indication for film reorganization with the IL anions slightly further away from and the cations closer to the Pt interface, due to higher mobility within the IL/D_2_O film.

After confirming the intactness of the IL between 100 and 150 K, we now address the water signal. After dosing at 137–140 K and cooling to ≈100 K, the initial O 1*s* D_2_O intensities were 73 ± 2 kcps for the 100, 130 and 150 K dataset. Notably, no attenuation of the D_2_O signal was observed upon IL deposition. Following IL dosing, the D_2_O intensities at 100 K were largely unchanged. This further suggests that the IL is not purely adsorbed on top of the water layer, but rather undergoes a co‐adsorption process (mixed IL‐water layer). Heating to 130 K leads to a minor decrease of the O_D2O_ intensity to 70 kcps. Further heating to 150 K leads to almost complete disappearance of the O_D2O_ signal.

#### 3.3.3 Orientation of 0.5 ML [C_1_C_1_Im][Tf_2_N] on 1 WL D_2_O/Pt(111)

**FIGURE 5:**
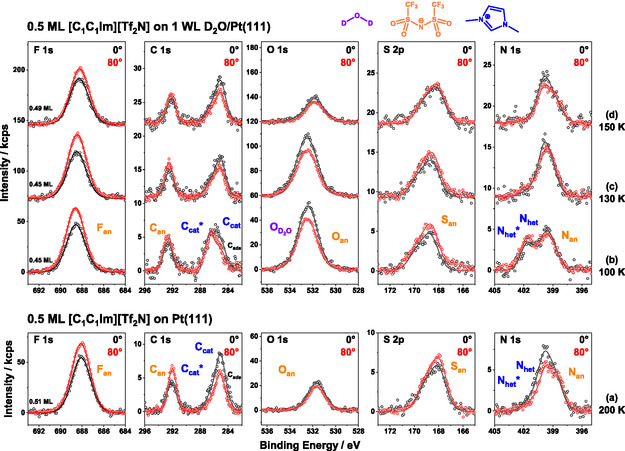
Isothermal XP‐spectra of 0.5 ML [C_1_C_1_Im][Tf_2_N] on (a) Pt(111) at 200 K (adapted from Ref. [21] with permission from PCCP Owner Societies) and on 1 WL D_2_O on Pt(111) at (b) 100, (c) 130, and (d) 150 K. Spectra were measured at 0° (black lines) and 80° (red lines). The data for 0° and the corresponding fitting is also shown in Figures 4 and S4. For better comparison, the 80° spectra have been scaled by factors of 0.56, 0.52, and 0.44 for 100, 130, and 150 K, respectively. At each temperature the same film was sequentially measured first at 0° and then at 80°.

As the next step, we examine the orientation of the IL on D_2_O/Pt(111) at 100, 130, and 150 K. To assess molecular orientation, we compare the more bulk‐sensitive 0° spectra (information depth: 6–9 nm, depending on kinetic energy of the respective core levels) with the more surface‐sensitive 80° spectra (information depth: 1–1.5 nm). The 0° spectra reflect more or less the average composition of the entire film, while the 80° spectra reveal increased signal intensities from species located near the vacuum/film interface and decreased intensities of those near the film/metal interface. The films were sequentially measured at 0° and 80°. Note that the 0° spectra were already shown in Figure 4. Given the overall increased intensity at 80° for film signals with our setup employing non‐focused X‐rays, all 80° spectra were normalized to the total IL plus D_2_O intensity, corrected by atomic sensitivity factors (ASF), obtained at 0°. This normalization resulted in scaling factors of 0.56 (100 K), 0.45 (130 K) and 0.49 (150 K), respectively. The spectra are plotted in Figure 5. For reference, the spectrum of the neat IL at 200 K (adapted from Ref. [21]) is also included. The 0° data is shown in black; the 80° data in red and the solid lines represent the fit envelope.

At 100 K (Figure 5b), we observe enhanced F_an_, C_an__,_ and S_an_ intensities at 80°, while N_an_ remains nearly unchanged between both angles, and the combined O_an_/O_D2O_ signal is decreased at 80°. This observation suggests that the anion orientation closely resembles that of the neat IL at 200 K—namely, the typical *cis*‐orientation of [Tf_2_N]^−^ with oxygen positioned near the D_2_O/Pt(111) interface, the CF_3_ groups orientated toward the vacuum, and the remaining elements in between [21]. The attenuation of the combined O_an_/O_D2O_ signal is significantly more pronounced than that of just the O_an_ signal in the neat IL (at 200 K), suggesting that D_2_O resides in close proximity to the Pt(111) surface. For the cation, the N_het_* signal is enhanced at 80°, whereas C_cat_ and N_het_ are attenuated, and C_cat_* remains largely unchanged. These angle‐dependent intensity changes are in line with IL cations in multiple binding motifs, which were deduced above from the binding energy differences between the asterisk (N_het_* and C_cat_*) and non‐asterisk components (N_het_ and C_cat_): Some cations (N_het_ and C_cat_) are likely adsorbed flat and in direct contact with the Pt(111) surface, while others (N_het_* and C_cat_*) reside further from the surface, possibly in a second layer. It also highlights a key difference compared with the neat IL (Figure 5a), where the ratio of asterisk to non‐asterisk component stays constant in 0° and 80°.

At 130 K (Figure 5c), these differences between neat IL and IL on D_2_O cation signals diminish. Cations that were previously located further from the Pt surface now appear to be in close contact with it, while the anion orientation remains largely unchanged. Notably, also the combined O_an_/O_D2O_ signal is unchanged, suggesting that some D_2_O remains at the Pt(111) surface, despite the cation adopting a configuration closer to substrate.

At 150 K (Figure 5d), the majority of water has desorbed, and all IL signals, including the O_an_/O_D2O_ signals, now closely resembles that of the neat IL.

#### 3.3.4 Conclusions for 0.5 ML [C_1_C_1_Im][Tf_2_N] on 1 WL D_2_O on Pt(111)

**FIGURE 6:**
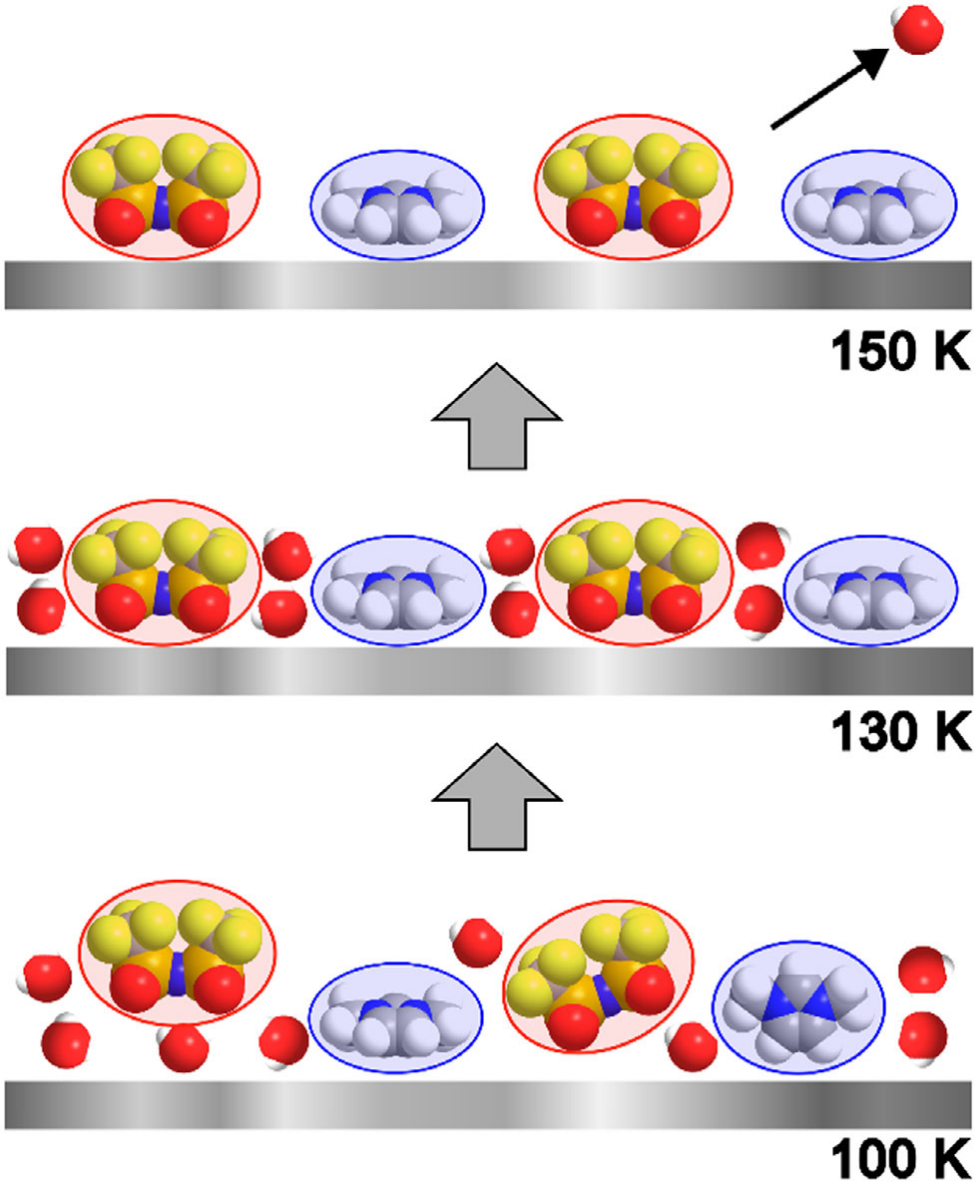
Schematic sketch of the proposed co‐adsorption structure of 0.5 ML [C_1_C_1_Im][Tf_2_N] on 1 WL D_2_O on Pt(111) at 100, 130, and 150 K.

Before we switch to the nitrile‐functionalized IL system, we want to summarize the observed behavior of [C_1_C_1_Im][Tf_2_N] on D_2_O/Pt(111). A schematic representation proposed for the most‐likely IL/D_2_O co‐adsorption structure at selected temperatures is shown in Figure 6 and serves as a guide for the following discussion. The investigated wetting layer (0.5 ML) [C_1_C_1_Im][Tf_2_N] films grow in a 2D layer‐by‐layer fashion on a D_2_O (CI) precovered Pt(111) surface at 100 K. The IL adsorbs partially in direct contact with the Pt(111) substrate and partially in the second layer. This is evident from the shifts of the IL peaks towards higher binding energy, and the significant changes in peak shape of particularly the nitrogen and carbon atoms of the aromatic system, due to the absence of the strong *π*‐interactions with the Pt(111) *d*‐bands. Since D_2_O initially forms a complete 2D film on the Pt surface and the O_D2O_ signal is not attenuated by the IL layer, we conclude that even at 100 K some of the pre‐dosed D_2_O must be displaced into the overlayer to accommodate the IL at the Pt(111) surface. This suggests a co‐adsorption structure where both IL and D_2_O are present at the interface as well as in the overlayer.

The [Tf_2_N]^−^ anions in the [C_1_C_1_Im][Tf_2_N]/D_2_O film after deposition at 100 K predominantly adopt the typical *cis*‐conformation with the oxygen atoms oriented toward the Pt(111) surface [21]. The cation exhibits multiple adsorption geometries: Some likely lie parallel to the surface, stabilized by *π*‐interactions, while others may adopt orientations aligned or tilted relative to the surface normal, in contrast to the pure IL wetting layer, where most cations lie flat. This indicates that despite the very strong interaction of the IL with the Pt(111) surface, the post‐deposited IL is only able to partly directly contact the surface, that is, a complete replacement of D_2_O at 100 K seems kinetically hindered.

Heating to 130 K increases the mobility of both IL and D_2_O, allowing the IL to rearrange and displace water further. This is reflected in a general shift of IL‐related signals toward lower binding energy. The similarity of the cationic peak structure to that of the neat IL at 200 K suggests that at 130 K, most of the IL cations in the IL/D_2_O film are fully in direct contact with the Pt(111) surface. However, the unchanged O 1*s* spectra at 80° indicate that water is not entirely pushed into the second layer. Compared to 100 K, a slightly different structure is formed at 130 K. The cation orientation appears more uniform, lying predominantly parallel to the surface, as suggested by the 80° data (Figure 5c) and supported by a decrease in C_cat_ intensity at 0° (Table 2). The orientation of the anion remains largely unaffected, but its XPS signals shift toward higher binding energy by 0.35–0.53 eV compared with the neat IL; these shifts indicate hydrogen bonding or dipole–dipole interactions with the co‐adsorbed water. Shifts in the cation signals could not be identified due to the constraints used to separate the asterisk (C_cat_* and N_het_*) from the non‐asterisk components (C_cat_ and N_het_). Notably, Broderick et al. observed binding energy shifts to higher values with increasing water content in bulk [BMIm][OAc] (=[C_4_C_1_Im][OAc]) [47]. They suggested hydrogen bonding as the origin of these shifts but refrained from further analysis without DFT calculations. In contrast, no binding energy shifts were reported for [BMIm][BF_4_] multilayers on anatase TiO_2_(101) upon water adsorption [48]. Notably, both studies reported a water‐induced rearrangement of the cations at the outermost surface. In bulk [BMIm][OAc], this manifested in an enrichment of alkyl carbon at the gas–liquid interface, attributed to the formation of nanoclusters above a certain water threshold. In the case of [BMIm][BF_4_], water adsorption led to a reorientation of the cations at the surface, with the polar headgroups pointing toward the gas phase/vacuum instead of the alkyl chains, facilitating interaction with adsorbed water.

In the [C_1_C_1_Im][Tf_2_N]/D_2_O/Pt(111) system presented here, the water‐metal interactions and water‐IL interactions are significantly weaker than IL‐metal interactions. As a result, the IL preferentially arranges itself close to the metal surface to maximize IL–substrate interaction. This rearrangement weakens the Pt–water interaction, leading to water desorption at ≈146 K. This temperature is ≈8 K lower than the desorption temperature of a pure D_2_O wetting layer (154 K). The data suggest that IL‐water interactions (water desorption temperature for mixed water/IL films: 146 K) are comparable to, or even weaker than, water–water interactions (desorption temperature of water multilayer: 149 K).

In our system, most D_2_O is desorbed at 150 K and the IL exhibits behavior similar to that of the neat IL, indicating no lasting influence from the initially co‐adsorbed water.

### 3.4 [C_3_CNC_1_Im][Tf_2_N] on D_2_O/Pt(111)

#### 3.4.1 Thermal Evolution

**FIGURE 7:**
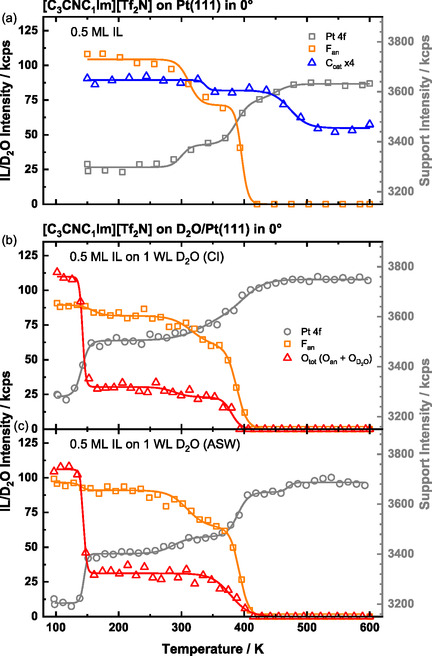
TPXPS of (a) neat 0.5 ML [C_3_CNC_1_Im][Tf_2_N] (adapted with permission from Ref. [36]. Copyright 2024 American Chemical Society) and 0.5 ML [C_3_CNC_1_Im][Tf_2_N] with 1 WL D_2_O deposited as (b) CI film or (c) ASW film on Pt(111). The Pt 4*f* (grey circles), C 1*s* (blue triangles), O 1*s* (red triangles), and F 1*s* (orange squares) core levels were measured while heating the sample with a heating rate of 2 K/s from ≈100 to 600 K. The intensity scale for the Pt 4*f* signals is shown on the right side.

After discussing the non‐functionalized IL [C_1_C_1_Im][Tf_2_N], we now turn to the adsorption/ orientation behavior and thermal stability of the nitrile‐functionalized IL [C_3_CNC_1_Im][Tf_2_N] on D_2_O/Pt(111); specifically, we also wanted to find out whether the adsorption behavior of the IL shows any differences on the CI‐D_2_O and ASW‐D_2_O layers. The thermal evolution of a 0.5 ML [C_3_CNC_1_Im][Tf_2_N] film is presented in Figure 7 for three different systems: (a) on Pt(111) (adapted from Ref. [36]), (b) on 1 WL D_2_O (CI) on Pt(111), and (c) on 1 WL D_2_O (ASW) on Pt(111). The preparation procedures follow those used for the nonfunctionalized IL described above; notably, the ASW film was deposited at 100 K, rather than at 137–140 K as for the crystalline ice. The exact IL coverages were 0.45 and 0.49 ML on top of 1 WL CI (Figure 7b) and 1 WL ASW (Figure 7c), respectively.

The reference plot of the neat [C_3_CNC_1_Im][Tf_2_N] on Pt(111) (Figure 7a) exhibit temperature dependencies that are similar to that of [C_1_C_1_Im][Tf_2_N] on Pt(111). Both ILs show stepwise decomposition, with the main difference being the enhanced thermal stability of [C_3_CNC_1_Im][Tf_2_N], where decomposition starts only above ≈280 K compared with ≈200 K for [C_1_C_1_Im][Tf_2_N] [21, 36]. When [C_3_CNC_1_Im][Tf_2_N] is post‐deposited on the pre‐adsorbed water layer (Figure 7b,c), its thermal evolution closely resembles that of [C_1_C_1_Im][Tf_2_N]/D_2_O(CI) on Pt(111) (Figure 3b). For the CI and ASW films, the combined O_an_/O_D2O_ signals decrease at 142 and 144 K, respectively, indicating water desorption. The F_an_ signal indicates that the remaining IL then decomposes in two distinct steps, consistent with the decrease of the F_an_ signal of the neat IL in Figure 7a. From this data, two conclusions can be drawn: (1) The water desorption temperature in the presence of the IL appears to be independent of the initial water structure, whether they are ordered (CI) or disordered (ASW), and (2) the desorption temperature of water is unaffected by the nitrile functionalization of the IL. In other words, both ILs have a comparable influence on water desorption behavior.

#### 3.4.2 Composition and Orientation

**FIGURE 8:**
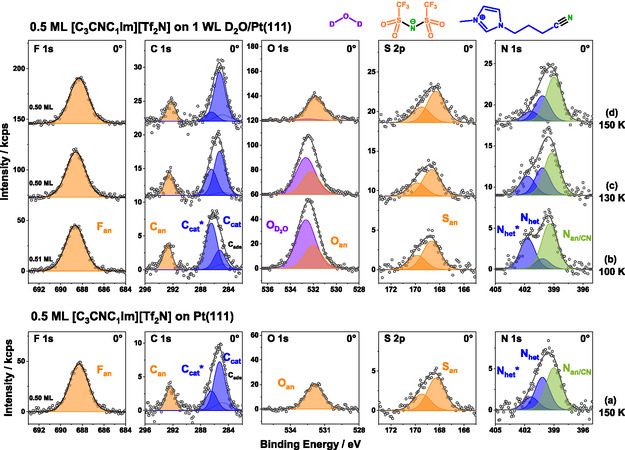
Isothermal XP‐spectra of 0.5 ML [C_3_CNC_1_Im][Tf_2_N] on (a) Pt(111) at 200 K (adapted with permission from Ref. [36]. Copyright 2024 American Chemical Society) and on 1 WL D_2_O on Pt(111) at (b) 100, (c) 130 and (d) 150 K, measured at 0° emission. For each temperature, a new sample was prepared. The peaks are color coded with blue for IL cation, orange for IL anion, green for the convoluted anion and cation, and purple for D_2_O‐related signals.

Also for the nitrile‐functionalized IL system [C_3_CNC_1_Im][Tf_2_N]/D_2_O on Pt(111), a complete set of XPS measurements were made at 100, 130, and 150 K and analyzed quantitatively. The preparation conditions were analogous to those used for the non‐functionalized IL [C_1_C_1_Im][Tf_2_N]. The 0° spectra are presented in Figure 8. The color coding follows that of [C_1_C_1_Im][Tf_2_N] in Figure 4, with the exception of the N_an/CN_ signal, which is colored green to reflect its dual origin from both the cationic nitrile group (N_CN_) and the anionic imidic nitrogen (N_an_) [36]. The corresponding peak positions and FWHM values are listed in Tables 1 and S1, respectively. As with [C_1_C_1_Im][Tf_2_N], two independent peaks were used to fit the cationic carbon signals (C_cat_ and C_cat_*) and the nitrogen atoms of the imidazolium ring (N_het_ and N_het_*) instead of single asymmetric peaks as it was previously done for the neat IL [36]. Note, that the C_cat_*/C_cat_ signals now includes three more carbon atoms from the longer alkyl‐chain and the nitrile group. For the quantitative analysis, the C_cat_*/C_cat_ and N_het_*/N_het_ peak pairs were summed up, respectively. The resulting relative atomic compositions at all three temperatures, shown in Table 3, agree well with the nominal values within our experimental uncertainty of ±15%. It should be noted that the systematic deviations of the F_an_ values (too large) and the C_cat_* + C_cat_ values (too small), relative to their nominal values, can partly be attributed to deviations in the ASF values at this coverage, as previously discussed (see Ref. [36]). Overall, the system [C_3_CNC_1_Im][Tf_2_N] on D_2_O/Pt(111) exhibits qualitatively the same behavior as observed for [C_1_C_1_Im][Tf_2_N] on D_2_O/Pt(111) and is thus interpreted in a similar way.

**TABLE 3:** Normalized (to 26 atoms) relative IL composition of 0.5 ML [C_3_CNC_1_Im][Tf_2_N] films with 1 WL D_2_O on Pt(111) at 100, 130, and 150 K. For C_cat_* and C_cat_, as well as N_het_* and N_het_ only the sum of the two contribution is given.

Composition		F_an_	C_an_	C_cat_* + C_cat_	O_an_	S_an_	N_het_* + N_het_	N_an/CN_	Σ	Σ_cation_	Σ_anion_
Nominal		6	2	8	4	2	2	2	26	11	15
**Pt(111), 0.5 ML, 1 WL D_2_O (CI)**											
**150 K**	**0°**	7.4	1.7	6.4	4.4	2.0	1.8	2.3	26	9.3	16.7
	**80°**	9.2	2.5	5.7	3.3	2.2	1.3	1.9	26	7.9	18.1
**130 K**	**0°**	7.0	1.8	7.3	4.2	1.6	2.1	2.1	26	10.5	15.5
	**80°**	8.9	2.4	5.7	3.5	2.2	1.6	1.7	26	8.1	17.9
**100 K**	**0°**	7.1	2.1	6.8	4.2	1.7	1.9	2.2	26	9.9	16.1
	**80°**	8.5	2.5	6.9	2.3	2.1	1.6	2.0	26	9.6	16.4

To address the orientation of the nitrile‐functionalized IL, we also performed measurements of the same preparations at 80°. The spectra are plotted in Figure S5, together with the 0° spectra, which were already presented in Figure 8. The 80° data is normalized to the 0° data and the scaling factors are 0.52 (100 K), 0.61 (130 K), and 0.43 (150 K), respectively. At 100 K, the behavior closely resembles that of the previously discussed [C_1_C_1_Im][Tf_2_N] on D_2_O/Pt(111) system. The F_an_, C_an_, and S_an_ intensities are enhanced at 80°, while N_an/CN_ remains nearly unchanged between 0° and 80°, and the combined O_an_/O_D2O_ signal is decreased at 80°. Although N_an/CN_ appears slightly attenuated in Figure S5, the fits shown in Figure S6 indicate that this effect primarily stems from the N_het_ component. This confirms that the anion adopts a *cis*‐configuration, similar to our observations discussed above for [C_1_C_1_Im][Tf_2_N]. Regarding the cation, C_cat_* and N_het_* are enhanced at 80°, while C_cat_ and N_het_ are attenuated, indicating distinct orientations, which again is in line with the interpretation given for [C_1_C_1_Im][Tf_2_N]. At 130 and 150 K, the orientation of the IL remains similar to that at 100K, with the observed small changes in the cationic species following the same trend as observed for [C_1_C_1_Im][Tf_2_N]. By 150 K, most of the D_2_O has desorbed and the spectra now closely resemble those of the neat IL at the same temperature [36].

#### 3.4.3 Comparison 0.5 ML [C_3_CNC_1_Im][Tf_2_N] and 0.5 ML [C_1_C_1_Im][Tf_2_N] on 1 WL D_2_O on Pt(111)

**FIGURE 9:**
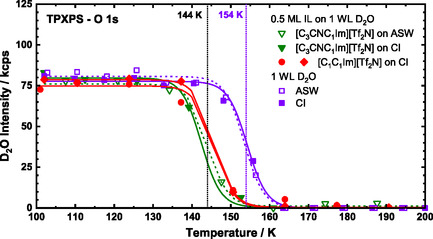
TPXPS of 1 WL D_2_O (purple), 0.5 ML [C_3_CNC_1_Im][Tf_2_N] on 1 WL D_2_O (green) and 0.5 ML [C_1_C_1_Im][Tf_2_N] on 1 WL D_2_O (red) on Pt(111). The red circles represent the data already shown in Figure 3b, while the red diamonds represent an additional dataset. The water layers have been prepared either at 100 K (ASW – layers) or at 137−140 K (CI layers); for details, see text. The solid lines serve as guide for the eye; the vertical dotted lines indicate the averaged desorption temperatures of 154 K for D_2_O (CI and ASW layers) and 144 K for the ILs on D_2_O (averaged over all IL preparations: 142 and 144 K for [C_3_CNC_1_Im][Tf_2_N] (green), and 146 K for [C_1_C_1_Im][Tf_2_N] (red)), as determined from the inflection points (IP). Note that for better comparison, a constant IL O_an_ signal intensity of ≈30 to 35 kcps was subtracted from the joint IL/water O 1*s* intensity for the IL/D_2_O layers.

The thermal evolution and structural orientation of [C_3_CNC_1_Im][Tf_2_N] shows qualitative agreement with [C_1_C_1_Im][Tf_2_N]. Between 100 and 150 K, both ILs exhibit similar behavior on D_2_O‐covered Pt(111). To highlight these similarities, Figure 9 presents the O 1*s* signals from the TPXP spectra of the two IL systems alongside that of pure water on Pt(111). For better comparison of the temperature range below 200 K (that is, before IL decomposition starts), a constant IL O_an_ signal intensity of ≈30 to 35 kcps was subtracted from the joint IL/water O 1*s* intensity. Purple data points represent pure 1 WL D_2_O, red indicate [C_1_C_1_Im][Tf_2_N] (0.5 ML) on 1 WL D_2_O and green denote [C_3_CNC_1_Im][Tf_2_N] (0.5 ML) on 1 WL D_2_O. Open symbols combined with dashed lines correspond to ASW films, while closed symbols and solid lines represent CI films. Figure 9 highlights three key results: (1) The difference between the two ILs is negligible and the average D_2_O desorption temperature is 144 ± 2 K (IP), which corresponds to a desorption energy of 48.4 kJ/mol using the above mentioned simple Redhead analysis; (2) the presence of 0.5 ML of any of the ILs clearly lowers the water desorption temperature by 10 ± 2 K compared with pure D_2_O (154 K, corresponding to 51.8 kJ/mol); (3) the initial water structure—whether ordered (CI) or disordered (ASW)—has no effect on D_2_O desorption behavior (see also Figure S7 for comparison of isothermal XPS data of the ILs on CI and ASW at 100 K).

The lack of a significant difference in the adsorption behavior and the thermal evolution of D_2_O between [C_1_C_1_Im][Tf_2_N] and [C_3_CNC_1_Im][Tf_2_N] suggests that the functionality within the imidazolium cation side chain plays a minor role in governing IL‐water interactions under the studied conditions. However, an important role of the anions in IL/water interactions is indicated by molecular dynamics simulations of [C_4_C_1_Im]^+^‐based ILs with various anions ([Tf_2_N]^−^, [PF_6_]^−^ and [BF_4_]^−^) confined between graphene electrodes, where water consistently accumulated near the positively charged electrode due to interactions with the anion [49, 50]. Similarly, vibrational spectroscopy studies on charged gold electrodes revealed that water interacts more strongly with [Tf_2_N]^−^ rather than with the cation [C_4_C_1_Im]^+^ [51].

## 4 Conclusions

Ultrathin films of the nonfunctionalized IL [C_1_C_1_Im][Tf_2_N] and the functionalized IL [C_3_CNC_1_Im][Tf_2_N] were deposited onto pre‐adsorbed single layer or multilayer crystalline (CI) or amorphous (ASW) water films (D_2_O) on Pt(111) at 100 K. These model systems were characterized at various temperatures using angle‐resolved X‐ray photoelectron spectroscopy in order to study the impact of water in the context of solid catalyst with ionic liquid layer (SCILL) catalysis. The growth behavior of both ionic liquids was characterized using the attenuation of the underlying Pt support: on both, D_2_O‐covered and clean Pt(111) surfaces, the ILs initially exhibit 2D layer‐by‐layer growth forming a closed IL wetting layer at 0.5 ML IL coverage. The growth mode changes to moderate 3D growth at IL coverages around 0.8–1.0 ML for both ILs.

At IL coverages of 0.5 ML, both ILs show similar adsorption behavior on D_2_O/Pt(111). At 100 K, the IL partially displaces D_2_O from the Pt(111) surface, leading to a co‐adsorption structure in which the IL is partly in direct contact with the substrate and partly located in a second layer. The [Tf_2_N]^−^ anion adsorbs in *cis*‐configuration with the oxygen atoms oriented toward the surface and CF_3_‐groups pointing toward the vacuum. The cations adopt different binding motifs, likely including both parallel and tilted orientations relative to the surface.

Upon heating to 130 K, an increased mobility within the IL/D_2_O films facilitates further rearrangement. The IL increasingly occupies sites directly at the Pt(111) surface, while the imidazolium cations adopt a more uniform, parallel orientation. Water remains co‐adsorbed but interacts more weakly with the IL than with the metal surface, as is deduced from the by 10 ± 2 K lower desorption temperature (which equals a by 3.4 kJ/mol smaller desorption energy) of D_2_O from the IL/D_2_O films as compared with the desorption of a D_2_O wetting layer on Pt(111) in the absence of the IL.

When comparing the functionalized and non‐functionalized ILs co‐adsorbed with D_2_O, no significant differences in adsorption geometry or thermal stability were observed, indicating that the presence of a nitrile group in the cation does not notably affect the interfacial behavior under these conditions.

This surface science study demonstrates that the IL exhibits a stronger affinity for the Pt(111) surface than water, leading to displacement of water from the metal–liquid interface. This suggests that in Supported Ionic Liquid Layer (SCILL) systems, catalytic reactions at the metal surface are unlikely to be significantly influenced by water contaminants at the interface. However, the role of water dissolved in the bulk IL phase may still be relevant.

## Supporting Information

Additional supporting information can be found online in the Supporting Information section. **Supporting Fig. S1:** Inverted LEED images of 3.1 WL D_2_O (ASW) on Pt(111), dosed and measured at (a) 100 K, heated to (b) 130 K, (c) 140 K and (d) 150 K. At 100 K, the amorphous water film does not give a distinct LEED pattern, only the 1x1 pattern from the underlying Pt(111)‐surface is visible. At 130 K and 140 K, the film reorganizes to form crystalline ice (CI) with a dim $\sqrt{3}x\sqrt{3}R30^{{}^{\circ}}$ structure. The $\sqrt{37}x\sqrt{37}R25.3^{{}^{\circ}}$ and $\sqrt{39}x\sqrt{39}R16.1^{{}^{\circ}}$ structures reported in literature were not observed, which is attributed to beam induced restructuring, as has been reported in Ref. [1]. At 150 K, the water completely desorbed leaving only the Pt(111) 1x1 structure. Image (a) was measured with a beam energy of 62 eV and (b)‐(d) with 65 eV. **Supporting Fig. S2:** F/O ratios obtained from the TPXPS of [C_1_C_1_Im][Tf_2_N] on D_2_O/Pt(111) in Figure 3b‐d. The dashed line indicates the F/O ratio of 2.8, independently obtained from the spectra of the neat ILs (see below), which was applied to constrain O_an_ when fitting the isothermal spectra. The IL/D_2_O TPXPS data shows a constant F/O ratio at this value between ∼160 and ∼270 K, indicating a parallel decrease of the anion‐related signals as decomposition starts above ∼200 K. **Supporting Fig. S3:** Schematic sketch of an alternative structure (to that shown in Figure 6) of 0.5 ML [C_1_C_1_Im][Tf_2_N] on 1 WL D_2_O on Pt(111) at 100, 130 and 150 K. At 100 K, the IL and D_2_O form clusters, which then convert to a co‐adsorption structure at 130 K. **Supporting Fig. S4:** Isothermal XP‐spectra of 0.5 ML [C_1_C_1_Im][Tf_2_N] on Pt(111) at 200 K (adapted from Ref. ^3^ with permission from PCCP Owner Societies), and on 1 WL D_2_O on Pt(111) at 100, 130 and 150 K, measured at 0° (black datapoints) and 80° (red datapoints) emission. For each temperature, a new sample was prepared, which was sequentially measured, first at 0° and then at 80. The peaks are colour coded with blue for IL cation, orange for IL anion and purple for D_2_O‐related signals. For better comparison, the 80° spectra have been scaled by factors of 0.56, 0.52, and 0.44 for 100, 130, and 150 K, respectively. The data was also shown in Figure 4 and 5. The full width at half maximum (FWHM), binding energies and quantitative analysis of the fits are listed in Table S1, 1 and 2, respectively. **Supporting Fig. S5:** Isothermal XP‐spectra of 0.5 ML [C_3_CNC_1_Im][Tf_2_N] on (a) Pt(111) at 200 K (adapted with permission from Ref. ^[4]^. Copyright 2024 American Chemical Society) and 1 WL D_2_O on Pt(111) at (b) 100 K, (c) 130 K and (d) 150 K. Spectra were measured at 0° (black lines) and 80° (red lines). The data for 0° and the corresponding fitting is also shown in Figure S6. For better comparison, the 80° spectra have been scaled by factors of 0.52, 0.61, and 0.43 for 100, 130, and 150 K, respectively. At each temperature the same film was sequentially measured, first at 0° and then at 80°. **Supporting Fig. S6:** Isothermal XP‐spectra of 0.5 ML [C_3_CNC_1_Im][Tf_2_N] on Pt(111) at 150 K (adapted with permission from Ref. ^[4]^. Copyright 2024 American Chemical Society), and on 1 WL D_2_O on Pt(111) at 100, 130 and 150 K, measured at 0° (black datapoints) and 80° (red datapoints) emission. For each temperature, a new sample was prepared, which was sequentially measured first at 0° and then at 80. The peaks are colour‐coded with blue for IL cation, orange for IL anion, green for the convoluted anion and cation and purple for D_2_O‐related signals. For better comparison, the 80° spectra have been scaled by factors of 0.52, 0.61, and 0.43 for 100, 130, and 150 K, respectively. The data was also shown in Figure 8 and S5. The full width at half maximum (FWHM), binding energies and quantitative analysis of the fits are listed in Table S1, 1 and 3, respectively. **Supporting Fig. S7:** Isothermal XP‐spectra of 0.51 (black datapoints) and 0.56 ML (blue datapoints) [C_3_CNC_1_Im][Tf_2_N] at 100 K, onto 1 WL D_2_O deposited as CI film (black datapoints) or ASW film (blue datapoints) on Pt(111). Each preparation, CI and ASW, was sequentially measured first at (a) 0° and then at (b) 80°. The 80° spectra have been scaled by factors of 0.50 and 0.52 for the ASW and CI film, respectively. The black data is also shown in Figure 8, S5 and S6. The spectra reveal no significant differences in peak shape or position between CI and ASW films at 100 K. **Supporting Table S1:** full width at half maximum (FWHM) values for wetting and sub‐wetting layer coverages, that is ≤ 0.5 ML IL and ≤ 1 WL D_2_O, of neat IL, IL/D_2_O (CI) and neat D_2_O (CI).

## Funding

This work was supported by the Deutsche Forschungsgemeinschaft (Grant: 431791331 ‐ SFB 1452).

## Conflicts of Interest

The authors declare no conflicts of interest.

## Supporting information

Supplementary Material

## Data Availability

The source data used in this paper will be available on Zenodo upon publication (https://doi.org/10.5281/zenodo.16919327).
