# Supplementary material for: Water Co‐Adsorption in Ultrathin Films of Ionic Liquids on Pt(111)
Source: ChemistryOpen. 2025 Nov 26;15(4):e202500571. doi: 10.1002/open.202500571 (PMC13052209; doi:10.1002/open.202500571)
Supplement: Supplementary file 1 — Supplementary Material [file OPEN-15-e202500571-s001.pdf]

SUPPORTING INFORMATION

Water Co-adsorption in Ultrathin Films of Ionic Liquids on Pt(111)

Timo Talwar, Hans-Peter Steinrück*, Florian Maier*

Lehrstuhl für Physikalische Chemie 2, Friedrich-Alexander-Universität Erlangen-Nürnberg, Egerlandstr. 3, 91058 Erlangen, Germany

Content:

- **Figure S1**: LEED images of D_2O/Pt(111) at different temperatures
- **Table S1**: FWHM values used for wetting and sub-wetting layer films
- **D_2O-molecules/IL-ion-pair ratio calculation**
- **Figure S2**: F/O ratios from the TPXP spectra in Figure 3b-d.
- **F_{an}/O_{an} ratios** in $[C_3CNC_1Im][Tf_2N]$ and $[C_1C_1Im][Tf_2N]$ on Pt(111)
- **Figure S3**: schematic sketch of IL/water cluster formation
- **Figure S4**: Isothermal spectra of $[C_1C_1Im][Tf_2N]$ at 0° and 80° and their corresponding fits
- **Figure S5**: Comparison of 0° and 80° isothermal data of $[C_3CNC_1Im][Tf_2N]/D_2O$/Pt(111)
- **Figure S6**: Isothermal spectra of $[C_3CNC_1Im][Tf_2N]$ at 0° and 80° and their corresponding fits
- **Figure S7**: Comparison of IL on ASW and CI films at 100 K for $[C_3CNC_1Im][Tf_2N]/D_2O$/ Pt(111)

Corresponding Authors:
Florian Maier (Florian.Maier@fau.de), Hans-Peter Steinrück (Hans-Peter.Steinrueck@fau.de)

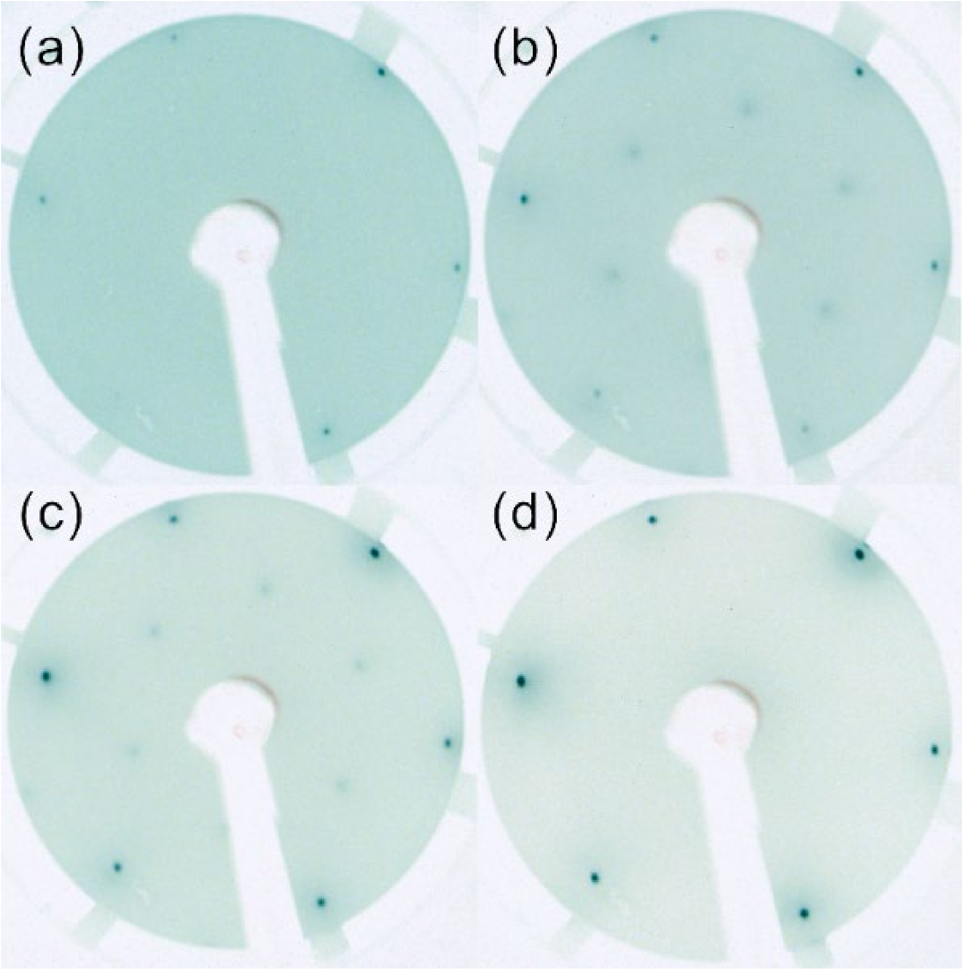

Figure S1: Inverted LEED images of 3.1 WL D_2O (ASW) on Pt(111), dosed and measured at (a) 100 K, heated to (b) 130 K, (c) 140 K and (d) 150 K. At 100 K, the amorphous water film does not give a distinct LEED pattern, only the 1x1 pattern from the underlying Pt(111)-surface is visible. At 130 K and 140 K, the film reorganizes to form crystalline ice (CI) with a dim $\sqrt{3}x\sqrt{3}R30°$ structure. The $\sqrt{37}x\sqrt{37}R25.3°$ and $\sqrt{39}x\sqrt{39}R16.1°$ structures reported in literature were not observed, which is attributed to beam induced restructuring, as has been reported in Ref. [1]. At 150 K, the water completely desorbed leaving only the Pt(111) 1x1 structure. Image (a) was measured with a beam energy of 62 eV and (b)-(d) with 65 eV.

Table S1: full width at half maximum (FWHM) values for wetting and sub-wetting layer coverages, that is ≤ 0.5 ML IL and ≤ 1 WL D_2O, of neat IL, IL/D_2O (CI) and neat D_2O (CI).

FWHM [eV]	F_{an}	C_{an}	C_{cat}*	C_{cat}	O_{an}	S_{an}	N_{het}*	N_{het}	$N_{an/CN}$/N_{an}	O_{D2O}
1 WL D_2O	/	/	/	/	/	/	/	/	/	1.95
0.5 ML [C_1C_1Im][Tf_2N]	2.04	1.48	1.67	1.67	1.82	1.53	1.60	1.60	1.70	/
0.5 ML [C_3CNC_1Im][Tf_2N]	2.06	1.48	1.92	1.92	1.76	1.70	1.80	1.80	1.90	/

Calculated D_2O/IL ratio from XPS O 1s signal intensities

The average IL oxygen signal (O_{an}) intensity obtained from the isothermal experiments at 100 and 130 K (partially shown in Figures 4, 5 and S3) is ~37 kcps, while the average water signal (O_{D2O}) intensity is ~73 kcps. This corresponds to a water-to-IL intensity ratio of two. Considering that one IL ion pair contains four oxygen atoms in the $[Tf_2N]^-$ anion, we obtain the water-to-IL ratio of eight water molecules per IL ion pair.

Calculated D_2O/IL ratio from literature values

The water to IL ratio can also be estimated from the corresponding surface densities of water and IL wetting layers on Pt(111).

Water density

The water density on Pt(111) is derived from the $\sqrt{3}x\sqrt{3}R30°$ LEED structure, with a unit cell area of

$$A_{\sqrt{3}x\sqrt{3}R30°} = 3xA_{Pt(111)1x1} = 0.20\,nm^2$$

where

$$A_{Pt(111)1x1} = {d_{Pt-Pt}}^2 * \frac{\sqrt{3}}{2} = (0.277\,nm)^2 * \frac{\sqrt{3}}{2} = 0.0664\,nm^2$$

Since the unit cell contains two water molecules, the corresponding surface density is:

$$\rho_{D_2O} = 2 * \frac{1\,molecule}{0.20\,nm^2} = 10\,\frac{molecule}{nm^2} = 10\,\frac{oxygen\ atoms}{nm^2}$$

$[C_1C_1Im][Tf_2N]$ density

For $[C_1C_1Im][Tf_2N]$ on Pt(111), the unit cell area is

$$A_{IL} = 0.81\,\frac{nm^2}{ion{-}pair},$$

Corresponding to a density of

$$\rho_{IL} = 1.22\,\frac{ion{-}pair}{nm^2},$$

as obtained from AFM/STM measurements.[2] Since each ion pair contains four oxygen in the $[Tf_2N]^-$ anion, this yields

$$\rho_{IL} = 4 * 1.22\,\frac{oxygen\ atoms}{nm^2} \approx 5\,\frac{oxygen\ atoms}{nm^2}.$$

The resulting O 1s intensity ratio of water to IL is therefore

$$\frac{O_{D_2O}}{O_{an}} = \frac{10}{5} = 2,$$

and the D_2O-to-IL ion-pair ratio is

$$\frac{\rho_{D_2O}}{\rho_{IL}} = \frac{10}{1.22} \approx 8\,\frac{D_2O{-}molecules}{IL{-}ion{-}pair}.$$

These values are in excellent agreement with those obtained from the XPS signal intensities.

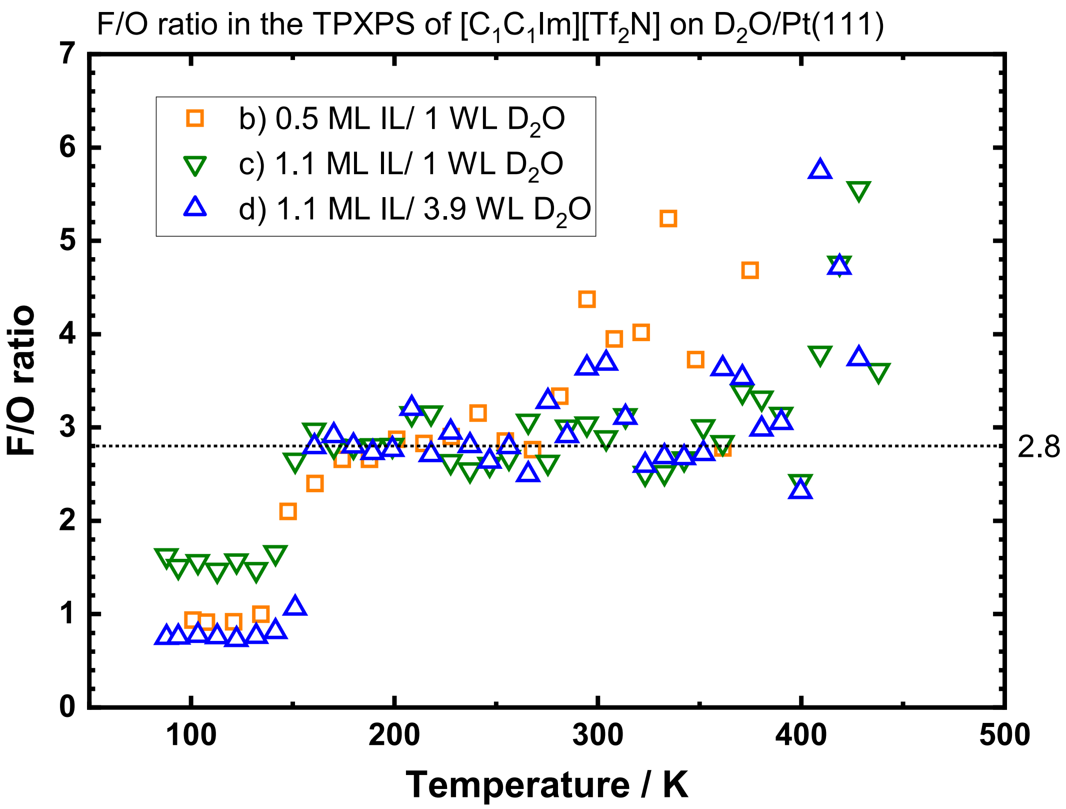

Figure S2: F/O ratios obtained from the TPXPS of $[C_1C_1Im][Tf_2N]$ on D_2O/Pt(111) in Figure 3b-d. The dashed line indicates the F/O ratio of 2.8, independently obtained from the spectra of the neat ILs (see below), which was applied to constrain O_{an} when fitting the isothermal spectra. The IL/D_2O TPXPS data shows a constant F/O ratio at this value between ~160 and ~270 K, indicating a parallel decrease of the anion-related signals as decomposition starts above ~200 K.

F_{an}/O_{an} ratios in $[C_3CNC_1Im][Tf_2N]$ and $[C_1C_1Im][Tf_2N]$ on Pt(111)

The F_{an}/O_{an} ratio was calculated based on the uncorrected peak areas of the corresponding F 1s and O 1s signals of 0.5 ML neat IL on Pt(111). For $[C_1C_1Im][Tf_2N]$ the F_{an}/O_{an} ratios are 2.52, 2.95 and 2.80 at 100, 150 and 200 K, respectively. The corresponding spectra for 100 and 200 K are shown in Ref.[3]. For $[C_3CNC_1Im][Tf_2N]$ the F_{an}/O_{an} ratios are 2.98 and 2.67 at 100 and 150 K, respectively. The corresponding spectra for 150 K are shown in Ref.[4]. Since $[C_1C_1Im][Tf_2N]$ starts decomposing above ~200 K and the temperature of interest is in between 100 and 200 K, the peak ratios at higher temperatures were not accounted for. The herein used average F_{an}/O_{an} ratio from both ILs is 2.8 (rounded from 2.78).

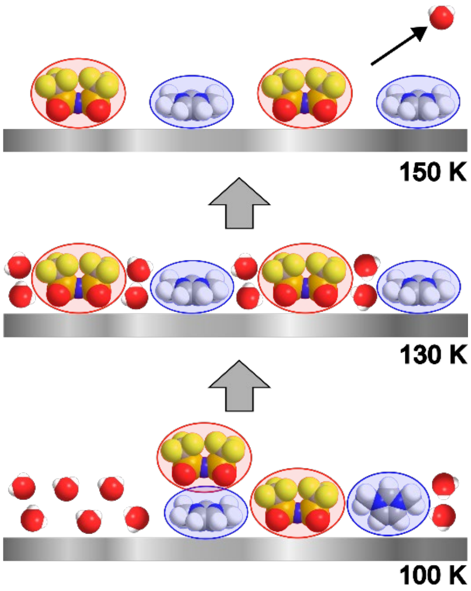

Figure S3: Schematic sketch of an alternative structure (to that shown in Figure 6) of 0.5 ML $[C_1C_1Im][Tf_2N]$ on 1 WL D_2O on Pt(111) at 100, 130 and 150 K. At 100 K, the IL and D_2O form clusters, which then convert to a co-adsorption structure at 130 K.

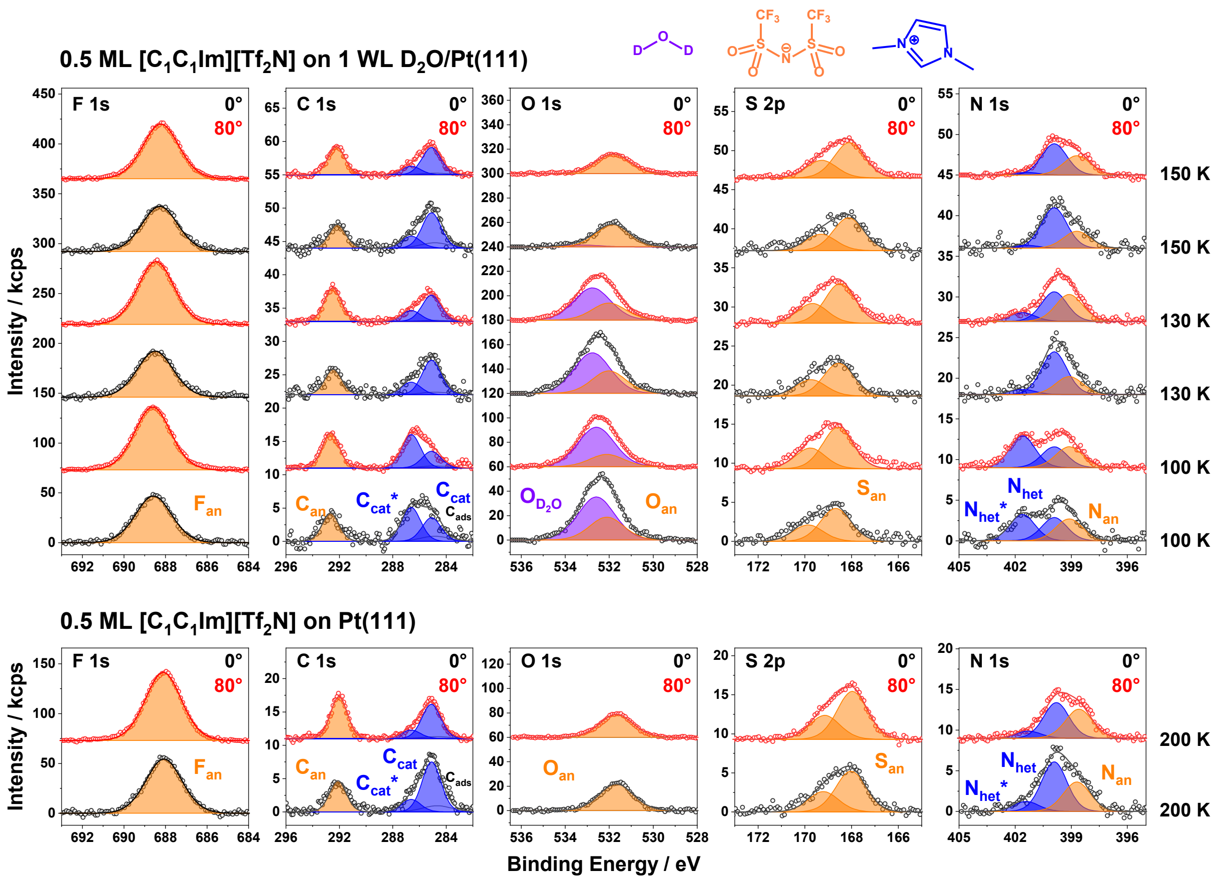

Figure S4: Isothermal XP-spectra of 0.5 ML $[C_1C_1Im][Tf_2N]$ on Pt(111) at 200 K (adapted from Ref. [3] with permission from PCCP Owner Societies), and on 1 WL D_2O on Pt(111) at 100, 130 and 150 K, measured at 0° (black datapoints) and 80° (red datapoints) emission. For each temperature, a new sample was prepared, which was sequentially measured, first at 0° and then at 80. The peaks are colour coded with blue for IL cation, orange for IL anion and purple for D_2O-related signals. For better comparison, the 80° spectra have been scaled by factors of 0.56, 0.52, and 0.44 for 100, 130, and 150 K, respectively. The data was also shown in Figure 4 and 5. The full width at half maximum (FWHM), binding energies and quantitative analysis of the fits are listed in Table S1, 1 and 2, respectively.

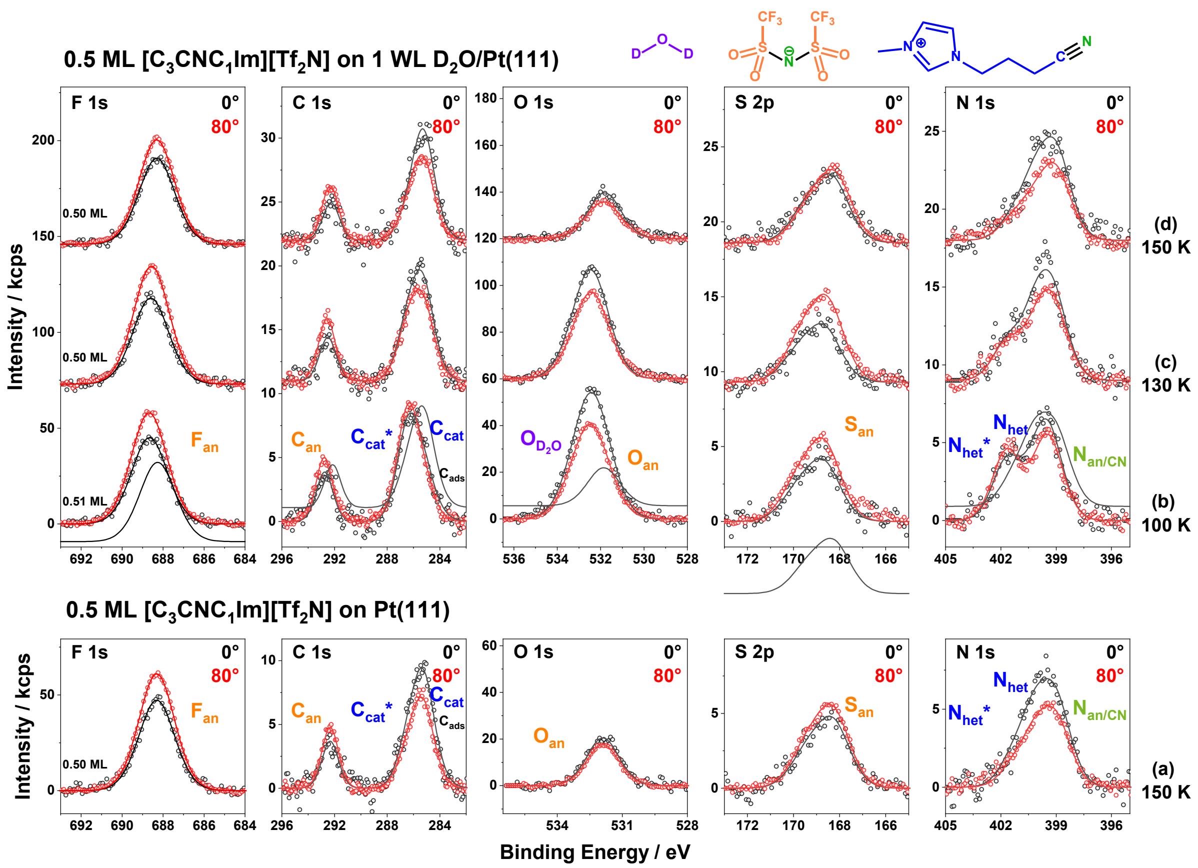

Figure S5: Isothermal XP-spectra of 0.5 ML [C_3CNC_1Im][Tf_2N] on (a) Pt(111) at 200 K (adapted with permission from Ref. [4]. Copyright 2024 American Chemical Society) and 1 WL D_2O on Pt(111) at (b) 100 K, (c) 130 K and (d) 150 K. Spectra were measured at 0° (black lines) and 80° (red lines). The data for 0° and the corresponding fitting is also shown in Figure S6. For better comparison, the 80° spectra have been scaled by factors of 0.52, 0.61, and 0.43 for 100, 130, and 150 K, respectively. At each temperature the same film was sequentially measured, first at 0° and then at 80°.

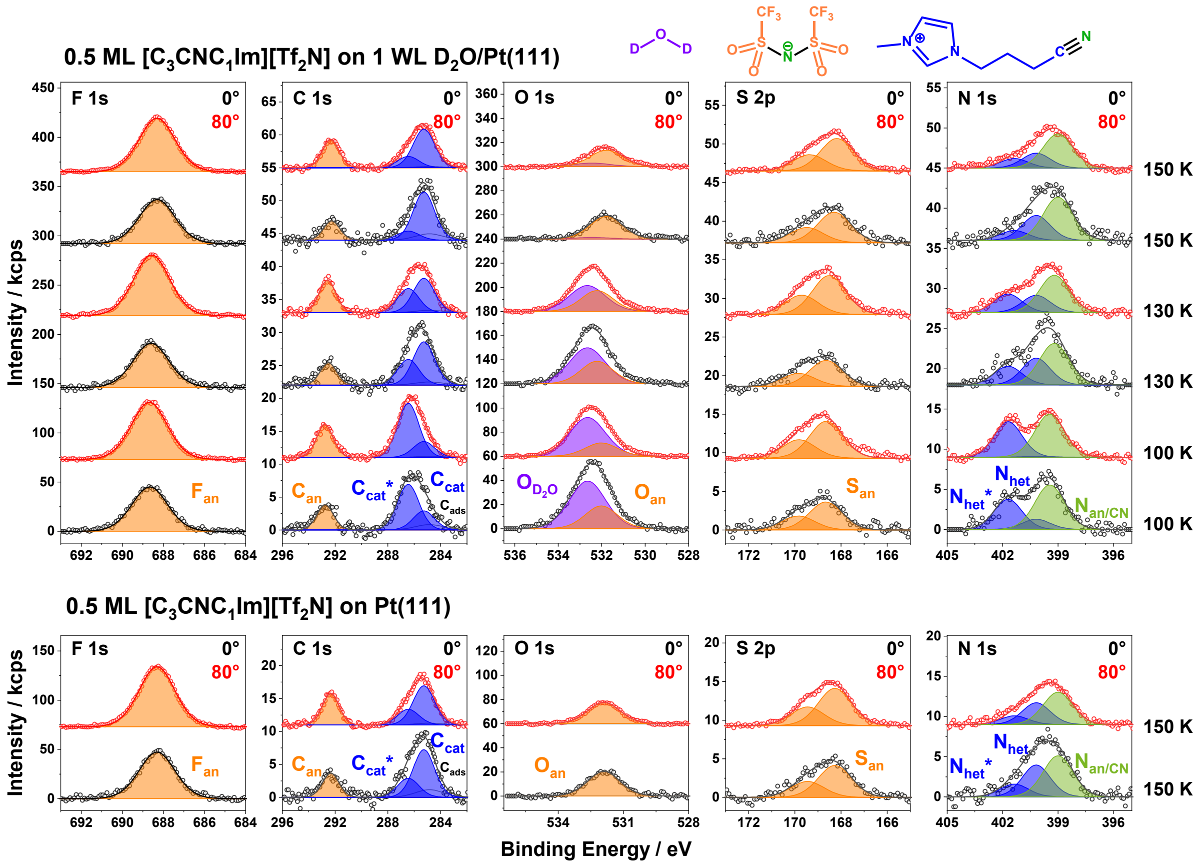

Figure S6: Isothermal XP-spectra of 0.5 ML [C_3CNC_1Im][Tf_2N] on Pt(111) at 150 K (adapted with permission from Ref. [4]. Copyright 2024 American Chemical Society), and on 1 WL D_2O on Pt(111) at 100, 130 and 150 K, measured at 0° (black datapoints) and 80° (red datapoints) emission. For each temperature, a new sample was prepared, which was sequentially measured first at 0° and then at 80. The peaks are colour-coded with blue for IL cation, orange for IL anion, green for the convoluted anion and cation and purple for D_2O-related signals. For better comparison, the 80° spectra have been scaled by factors of 0.52, 0.61, and 0.43 for 100, 130, and 150 K, respectively. The data was also shown in Figure 8 and S5. The full width at half maximum (FWHM), binding energies and quantitative analysis of the fits are listed in Table S1, 1 and 3, respectively.

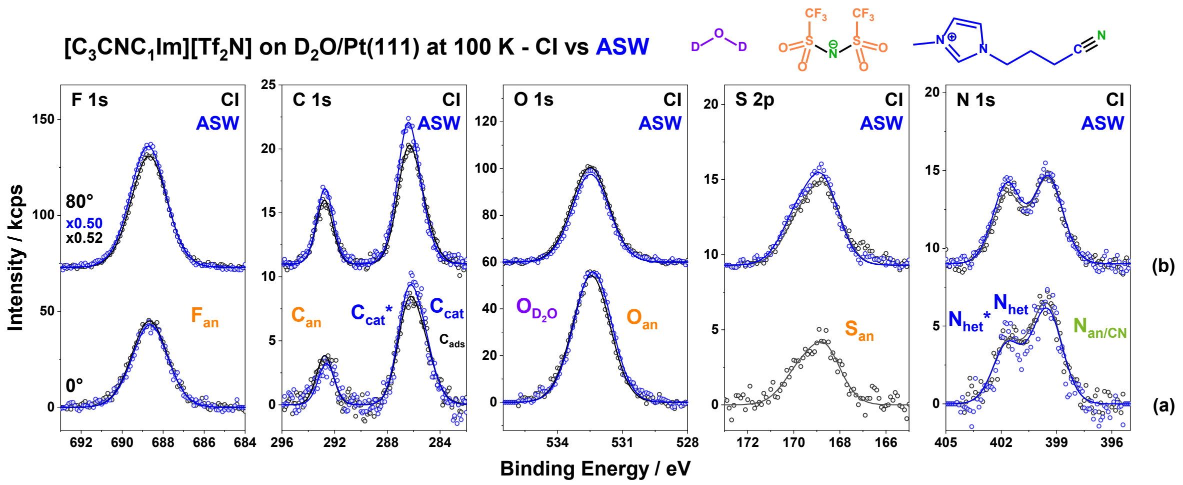

Figure S7: Isothermal XP-spectra of 0.51 (black datapoints) and 0.56 ML (blue datapoints) $[C_3CNC_1Im][Tf_2N]$ at 100 K, onto 1 WL D_2O deposited as CI film (black datapoints) or ASW film (blue datapoints) on Pt(111). Each preparation, CI and ASW, was sequentially measured first at (a) 0° and then at (b) 80°. The 80° spectra have been scaled by factors of 0.50 and 0.52 for the ASW and CI film, respectively. The black data is also shown in Figure 8, S5 and S6. The spectra reveal no significant differences in peak shape or position between CI and ASW films at 100 K.

References

[1] J. Harnett, S. Haq, A. Hodgson, "Electron induced restructuring of crystalline ice adsorbed on Pt(111)" *Surf. Sci.* **2003,** *528*, 15-19.

[2] A. Gezmis, T. Talwar, M. Meusel, A. Bayer, F. Maier, H.-P. Steinrück, "Flexible 2D Structure Formation of $[C_1C_1Im][Tf_2N]$ on Ag(111)" *ChemPhysChem* **2025,** *26* , e202500163.

[3] S. Massicot, A. Gezmis, T. Talwar, M. Meusel, S. Jaekel, R. Adhikari, L. Winter, C.C. Fernandez, A. Bayer, F. Maier, H.-P. Steinrück, "Adsorption and thermal evolution of $[C_1C_1Im][Tf_2N]$ on Pt(111)" *Phys. Chem. Chem. Phys.* **2023,** *25* , 27953-27966.

[4] T. Talwar, J. Barreto, C.C. Fernández, H.-P. Steinrück, F. Maier, "Ultrathin Films of a Nitrile-Functionalized Ionic Liquid $[C_3CNC_1Im][Tf_2N]$ on Au(111) and Pt(111): Adsorption, Growth, and Thermal Behavior" *Langmuir* **2024,** *40* , 27565-27578.
